# Synaptic dysregulation and hyperexcitability induced by intracellular amyloid beta oligomers

**DOI:** 10.1111/acel.13455

**Published:** 2021-08-19

**Authors:** Eduardo J. Fernandez‐Perez, Braulio Muñoz, Denisse A. Bascuñan, Christian Peters, Nicolas O. Riffo‐Lepe, Maria P. Espinoza, Peter J. Morgan, Caroline Filippi, Romain Bourboulou, Urmi Sengupta, Rakez Kayed, Jérôme Epsztein, Luis G. Aguayo

**Affiliations:** ^1^ Laboratory of Neurophysiology Department of Physiology Universidad de Concepción Concepción Chile; ^2^ Institute of Neurobiology of the Mediterranean Sea (INMED) Institut National de la Santé et de la Recherche Médicale (INSERM) U901, Aix-Marseille Université Marseille France; ^3^ Mitchell Center for Neurodegenerative Diseases University of Texas Medical Branch Galveston Texas USA; ^4^ Department of Neurology, Neuroscience and Cell Biology University of Texas Medical Branch Galveston Texas USA

**Keywords:** AMPA‐R, hyperexcitability, intracellular amyloid beta, nitric oxide, synaptic dysregulation

## Abstract

Intracellular amyloid beta oligomer (iAβo) accumulation and neuronal hyperexcitability are two crucial events at early stages of Alzheimer's disease (AD). However, to date, no mechanism linking iAβo with an increase in neuronal excitability has been reported. Here, the effects of human AD brain‐derived (h‐iAβo) and synthetic (iAβo) peptides on synaptic currents and action potential firing were investigated in hippocampal neurons. Starting from 500 pM, iAβo rapidly increased the frequency of synaptic currents and higher concentrations potentiated the AMPA receptor‐mediated current. Both effects were PKC‐dependent. Parallel recordings of synaptic currents and nitric oxide (NO)‐associated fluorescence showed that the increased frequency, related to pre‐synaptic release, was dependent on a NO‐mediated retrograde signaling. Moreover, increased synchronization in NO production was also observed in neurons neighboring those dialyzed with iAβo, indicating that iAβo can increase network excitability at a distance. Current‐clamp recordings suggested that iAβo increased neuronal excitability via AMPA‐driven synaptic activity without altering membrane intrinsic properties. These results strongly indicate that iAβo causes functional spreading of hyperexcitability through a synaptic‐driven mechanism and offers an important neuropathological significance to intracellular species in the initial stages of AD, which include brain hyperexcitability and seizures.

## Graphical abstract

In the presence of iAβo, PKC is activated (a), which in turn allows the production of nitric oxide (NO) at the post‐synaptic level, increasing the release of neurotransmitters (b) (evidenced as an increase in the frequency of mPSC). PKC also increases AMPA receptor current (c), increasing post‐synaptic depolarization and neuronal excitability (d). Finally, iAβo increases synchronization NO production in nearby neurons that do not have iAβo (e).

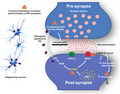

**Abbreviations**
AD: Alzheimer's disease
AMPA: α‐amino‐3‐hydroxy‐5‐methyl‐4‐isoxazolepropionic acid
APP: amyloid precursor protein
APs: action potentials
Aβ: amyloid‐β peptide
CLR: chelerythrine
eEPSC: evoked excitatory post‐synaptic currents
eIPSC: evoked inhibitory post‐synaptic currents
eNOS: endothelial nitric oxide synthase
h‐iAβo: human AD brain‐derived Aβ oligomers
iAβo: synthetic Intracellular Aβ oligomers
iNOS: inducible nitric oxide synthase
mEPSC: miniature excitatory post‐synaptic currents
mIPSC: miniature inhibitory post‐synaptic currents
mPSC: miniature post‐synaptic currents
Na_v_
: voltage‐dependent sodium channel
nNOS: neuronal nitric oxide synthase
NO: nitric oxide
NOS: nitric oxide synthase
PKC: protein kinase C
PLC: phospholipase C
PSEN1: presenilin‐1
TTX: tetrodotoxin
*V*_m_
: membrane potential

## 1 INTRODUCTION

Numerous studies reported that amyloid beta (Aβ) plays an important role in the synaptic dysfunction observed in Alzheimer's disease (AD) patients (Edwards, 2019; Selkoe & Hardy, 2016). Unfortunately, current therapeutic targets are focused on extracellular Aβ accumulation at a stage when the disease is well underway. However, there might be previous events in the pathology that are more important in the early pre‐clinical stages; thus, other mechanisms for the pathogenesis of AD need to be considered. For example, it was recently reported that prior to the formation of extracellular Aβ deposits and intracellular tau tangles in the human brain, there is an intracellular accumulation of soluble Aβ oligomers (iAβo) during AD, especially in vulnerable regions such as the entorhinal cortex and hippocampus (Welikovitch et al., 2018). Moreover, it has been suggested that amyloid plaques can result from iAβo accumulation, suggesting that extracellular deposits are not the exclusive origin of senile plaques (Pensalfini et al., 2014), thus highlighting the importance of iAβo as an early stage in the progression of the pathology. The presence of iAβo has also been associated with synaptic dysfunction in different AD mice models, which could play a key role in the cognitive deficit observed in this disease. For example, the 3xTg AD model develops intraneuronal accumulation of Aβ between 3 and 4 months of age (Oddo et al., 2003), a time when cognitive deficits are first detected and a stage with little, if any, presence of extracellular Aβ (Billings et al., 2005). Interestingly, the removal of iAβo with immunotherapy improves cognition in this model (Billings et al., 2005), and as the pathology reemerges, there is a reappearance of intraneuronal Aβ followed by the formation of extracellular amyloid plaques (Oddo et al., 2006). Thus, growing evidence supports intraneuronal accumulation of Aβ as an early event in the course of the pathology, and there are studies in at least seven other murine AD models (including 5xFAD, amyloid precursor protein (APP)/tau, APP751SL/PS1 KI, APPE693Δ and TBA2) (Iulita et al., 2014; Tomiyama et al., 2010; Wirths & Bayer, 2010) that also show the presence of intraneuronal Aβ prior to extracellular accumulation, independent of the mutation they carry.

Early intracellular Aβ accumulation is particularly interesting since recent evidence has shown that changes in neuronal activity could also be playing a fundamental role in the initial stages of Aβ pathology, predisposing the development of a synaptopathology postulated in the initial stages of AD. For example, subjects diagnosed in the early stages of prodromal AD (a very early form of AD) displayed increased neuronal activity in the hippocampus and cortex (Dickerson et al., 2005; Huijbers et al., 2015). Additionally, it was found that patients might exhibit increased neuronal excitability and even present a higher risk of seizures (Amatniek et al., 2006; Born, 2015; Hommet et al., 2008). Indeed, two independent studies demonstrated that seizures were present in ~10–20% of patients diagnosed with sporadic AD (Lozsadi & Larner, 2006; Mendez & Lim, 2003), and patients with familial AD with mutations in APP, presinilin‐1 (PS1), and presinilin‐2 (PS2) also exhibited an increased prevalence of seizures (Cabrejo et al., 2006; Marcon et al., 2004; Snider et al., 2005). It is still largely unknown how hyperexcitability can arise in an AD brain. Possible explanations for this phenomena include changes in excitatory/inhibitory balance (Bi et al., 2020; Hijazi et al., 2019; Vico Varela et al., 2019) and/or increase in intrinsic membrane excitability properties (Kellner et al., 2014). In fact, these early changes in excitability are in agreement with *in vitro* studies that showed epileptiform activity (Cuevas et al., 2011) and hyper‐synchronous neuronal activity following acute exposure to extracellular Aβ, as well as in several *in vivo* models that overexpress Aβ, exhibiting altered intrinsic excitability (Bezzina et al., 2015; Born et al., 2014; Brown et al., 2011; Davis et al., 2014; Marcantoni et al., 2014) and convulsive neuronal activity (Brown et al., 2011; Minkeviciene et al., 2009).

Since intraneuronal accumulation is an early event in the pathology, these studies suggest a potential link between iAβo and neuronal hyperexcitability. However, a potential cellular mechanism that might explain this potential association has not been reported. Here, we describe that h‐iAβo applied to the post‐synaptic neuron increased global synaptic transmission, excitability, and neuronal synchronization in *in vitro* and *in vivo* hippocampal models. Data show that the pre‐synaptic action of iAβo was primarily caused by a NO‐dependent retrograde signaling, while the post‐synaptic effect was mediated by the potentiation in α‐amino‐3‐hydroxy‐5‐methyl‐4‐isoxazolepropionic acid (AMPA) currents by a protein kinase C (PKC)‐dependent mechanism.

## 2 RESULTS

### 2.1 Effect of human‐derived iAβo in hippocampal neuronal activity

**FIGURE 1:**
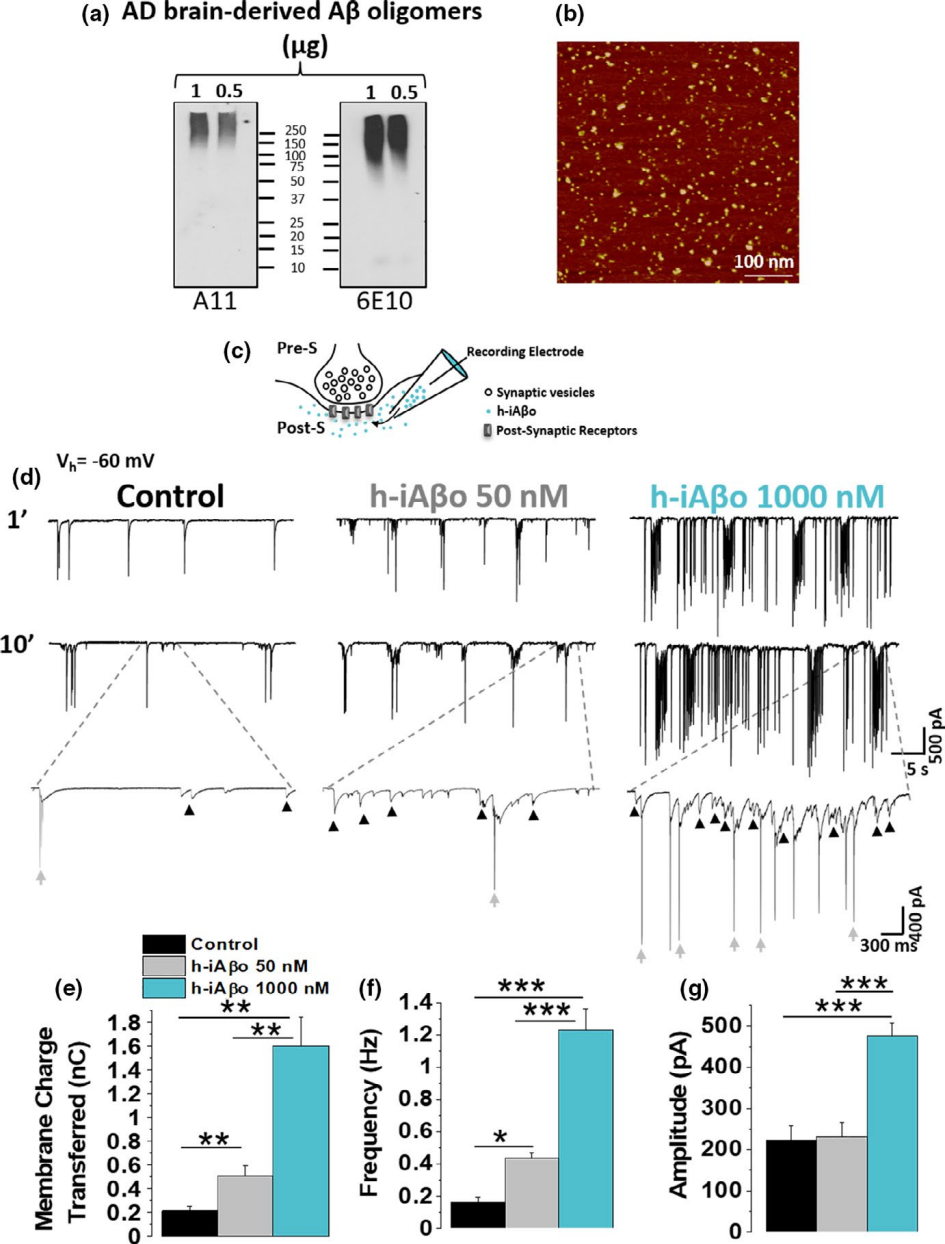
Intracellular human‐derived Aβ oligomers (h‐iAβo) increased the synaptic transmission and AP spike firing in hippocampal neurons *in vitro*. (a) Western blot analysis of AD brain‐derived Aβ oligomers with A11 and generic Aβ antibody 6E10 (1 µg = 250 µM, 0.5 µg = 125 µM). (b) AFM image of 0.5 µg (125 µM) AD brain‐derived Aβ oligomer. Scale bar 100 nm. (c) Schematic representation of synaptic recordings showing the pre‐synaptic (Pre‐S) and post‐synaptic (Post‐S) compartment, and the use of the patch electrode to dialyze the neuron with h‐iAβo and to record membrane currents (electrode does not represent actual size). (d) Representative synaptic current recordings [holding potential (*V*h) = −60 mV] obtained in primary hippocampal neurons (*in vitro*). Spontaneous synaptic currents (black arrowheads) and spikes in current recording mode (gray arrows) are observed. (e–g) Quantification of charge transferred (e) frequency (f) and amplitude (g) of post‐synaptic currents. Bars represent the average ± SEM for control (*n* = 6), h‐iAβo 50 nM (*n* = 7) and h‐iAβo 1000 nM (*n* = 8) cells. One‐Way Welch's ANOVA with Games–Howell *post hoc* test for (e): *F*(2, 9.91) = 24.04, *p* = 1.57E‐4. *p*‐values for *post hoc* test: control vs. h‐iAβo 50 nM: 5.68E‐3, control vs. h‐iAβo 1000 nM: 1.51E‐9 and h‐iAβo 50 nM vs. h‐iAβo 1000 nM: 6.76E‐3. One‐Way Welch's ANOVA with Games–Howell *post hoc* test for (f): *F*(2, 10.69) = 32.82, *p* = 2.73E‐5. p‐values for *post hoc* test: control vs. h‐iAβo 50 nM: 3.29E‐2, control vs. h‐iAβo 1000 nM: 1.17E‐4, h‐iAβo 50 nM vs. h‐iAβo 1000 nM: 3.80E‐4. One‐Way Welch's ANOVA with Games–Howell *post hoc* test for (g): *F*(2, 11.09) = 19.25, *p* = 2.47E‐4. *p*‐values for *post hoc* test: control vs. h‐iAβo 1000 nM: 6.94E‐04, h‐iAβo 50 nM vs. h‐iAβo 1000 nM: 4.67E‐4. * denotes *p* < 0.05, ** *p* < 0.01, *** *p* <0.001. For data with fluorescently labeled synthetic iAβo, see Figure S1

We examined the synaptic effects of human AD brain‐derived intracellular oligomers (h‐iAβo) using an electrophysiological approach. First, characterization of the biochemically purified Aβ species with WB analysis using A11 (anti‐amyloid oligomers) and 6E10 (generic anti‐Aβ) antibodies confirmed the presence of Aβo in this preparation (Figure 1a), and AFM imaging showed a homogeneously distributed population of sphere‐shaped oligomers ranging from 5 to 20 nm in size (Figure 1b). Using the intracellular solution, we delivered increasing concentrations (0, 50, and 1000 nM) of h‐iAβo while recording post‐synaptic currents in cultured hippocampal neurons (see scheme in Figure 1c). Control synaptic current recordings were made with an intracellular solution without h‐iAβo. Electrophysiological recordings showed the presence of spontaneous synaptic events (Figure 1d, arrows in blue) that had a stable response over a period of 10 min. Applying 50 nM h‐iAβo with the patch electrode augmented the presence of spontaneous synaptic currents interspersed in the recording (Figure 1d, arrowheads in middle trace). It was also possible to observe that part of the total activity was mediated by spikes in current recording mode, which also augmented in the presence of 50 nM h‐iAβo (Figure 1d, arrows in middle trace). The increase in activity was a concentration‐dependent phenomenon, given that there were more and larger amplitude events as the concentration of iAβo augmented from 50 to 1000 nM (the number of replicates was >6 neurons and is indicated in the legends). We integrated the area under the current trace for each condition and computed the charge transferred for the recorded cell (see calculation details in methods, Section 2.10). 1000 nM h‐iAβo significantly increased the charge transferred meaning that more current per unit of time was flowing through the membrane compared to control conditions (Figure 1e, *n *> 6 neurons). Interestingly, 50 and 1000 nM produced an increase in the frequency of the synaptic events (Figure 1f), but only 1000 nM augmented the amplitude (Figure 1g). To corroborate that the entry of Aβ to the neuron was rapid and to validate the method as an approximation to effectively deliver Aβo to the intraneuronal compartment, we repeated this experiment using a fluorescently labeled synthetic Aβo. The results confirmed that iAβo in the patch pipette was in fact rapidly delivered to the intracellular compartment, since the increase in synaptic activity was correlated with a temporal increase in fluorescence, with a *t*_1/2_ of 29 s (see Figure S1). Interestingly, the data obtained with human (Figure 1) and synthetic oligomers (Figure S1) produced very similar activation in these neurons.

### 2.2 Concentration‐dependent increase in miniature currents by iAβo

**FIGURE 2:**
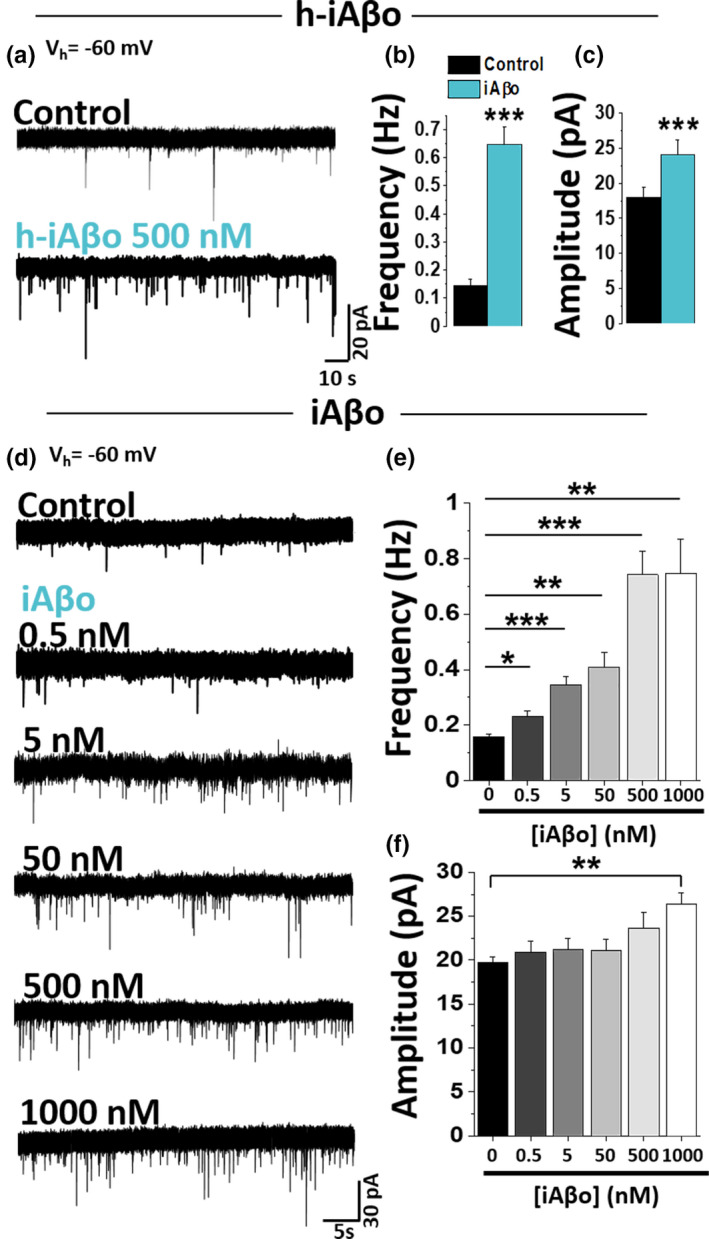
Effects of human and synthetic intracellular Aβ oligomers on the frequency and amplitude of miniature post‐synaptic currents *in vitro*. (a) Representative mPSC traces after applying 0.5 µM h‐iAβo (*V*_h_ = −60 mV). Recording of primary hippocampal neurons was obtained in the presence of TTX 500 nM. (b, c) mPSC frequency (b) and amplitude (c) quantification for control (*n* = 5 cells) and 500 nM h‐iAβo (*n* = 9 cells). (d) mPSC traces for control (*n* = 16) and after applying increasing concentrations of synthetic iAβo 0.5 nM (*n* = 14), 5 nM (*n* = 16), 50 nM (*n* = 16), 500 nM (*n* = 14), and 1000 nM (*n* = 16 cells). (e, f) mPSC frequency (e) and amplitude (f) for each of the conditions described in (d). Bar charts represent the average ± SEM. Unpaired Student's *t* test for (b) (*t*(12) = −5.845, *p* = 7.89E‐05) and unpaired Student's *t* test with Welch's correction for (c) (*t*(11.93) = −5.123, *p* = 2.56E‐04). One‐Way Welch's ANOVA with Games–Howell *post hoc* test for (e): *F*(5, 86) = 13.76, *p *= 7.15E‐10. *p*‐values for *post hoc* test: iAβo 0 nM vs. 0.5 nM: 4.57E‐2, iAβo 0 nM vs. 5 nM: 2.73E‐4, iAβo 0 nM vs. 50 nM: 2.33E‐3, iAβo 0 nM vs. 500 nM: 1.17E‐4, iAβo 0 nM vs. 1000 nM: 3.11E‐3. One‐Way Welch's ANOVA with Games–Howell *post hoc* test for (f): *F*(5, 86) = 3.73, *p *= 0.004. *p*‐values for *post hoc* test: iAβo 0 nM vs. 1000 nM: 2.01E‐3. * denotes *p* <0.05, ** *p* <0.01 and *** *p* <0.001. For control experiments, see Figures S2 and S3

It is accepted that in order to record synaptic currents at the post‐synaptic site, the release of neurotransmitters must occur at the pre‐synaptic terminal (Figure 1c). To quantify the effect of iAβo on actual synaptic transmission, we recorded miniature post‐synaptic currents (mPSC) in cultured neurons. Intracellular application of 500 nM of h‐iAβo was able to produce a rapid and significant increase in the frequency and amplitude of mPSC in cultured neurons (Figure 2a–c). This result was confirmed using several concentrations of the synthetic form of Aβ (iAβo) because it is much more available than the native h‐iAβo, thus allowing us to examine a large number of neurons. The data showed that in the presence of iAβo, there was a significant increase in the number of events as the concentration was increased, indicating a concentration‐dependent effect (Figure 2d). The effects of iAβo were more evident on the frequency of miniature events since statistically significant differences were already found at 0.5 nM of iAβo when compared to control conditions (Figure 2e). On the other hand, the effect of iAβo on the amplitude was only significant at 1000 nM (Figure 2f). Thus, the effects of low concentrations of human and synthetic oligomers are seen in the frequency of the synaptic events, but not in their amplitude. We performed several controls to eliminate potential confounds with the application of intracellular iAβo using the patch pipette. Using the same methodology, we first tested the reverse sequence Aβo and then the vehicle used to dissolve the peptide (see details of solvents used in Section 2.2). Under these conditions, no significant differences with respect to the control condition were found (Figure S2). We then determined the osmolarity of the internal solution under the different iAβo concentrations (Figure S2). Finally, we observed a significant attenuation in the presence of a specific antibody (A11) that recognizes oligomeric forms (Yoshiike et al., 2007) (Figure S3). In addition, we tested the effect of iAβo_40_ and iAβo_42_ isoforms and found that both increased the frequency, but not the amplitude of the synaptic events (Figure S4). These results showed that Aβo is quickly dialyzed into the neuron, and the effects are mediated by the peptide in a concentration‐dependent fashion.

### 2.3 Human iAβo affects excitatory/inhibitory (E/I) balance *in vitro*

**FIGURE 3:**
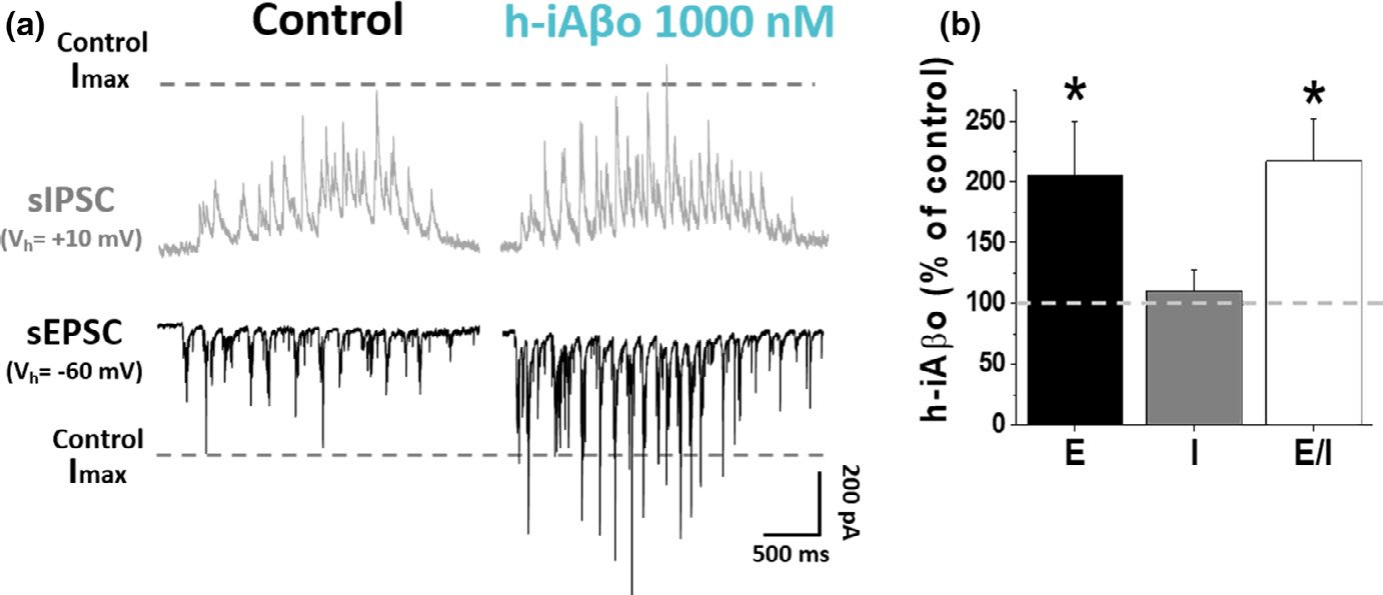
h‐iAβo induces a shift in excitatory/inhibitory (E/I) balance *in vitro*. (a) Spontaneous inhibitory (sIPSC) and excitatory (sEPSC) post‐synaptic recordings obtained in the absence (control) and the presence of 1 µM h‐iAβo. Recording of primary hippocampal neurons was obtained in the presence of 5 mM QX‐314 and using a “low chloride internal solution” (details in Section 2.4.1). In this condition, reverse potential for Cl^−^ was ≈ −66 mV, allowing us to record sEPSC at a *V*_h_ of −60 mV and sIPSC at +10 mV. Segmented lines show the maximum amplitude observed for sEPSC and sIPSC in control condition (Control Imax). (b) Charge transferred for sEPSC (E) (unpaired Student's *t* test: *t*(11) = −2.75, *p* = 1.86E‐2), charge transferred for sIPSC (I), and E/I ratio for each condition (unpaired Student's *t* test: *t*(11) = −2.73, *p* = 1.93E‐2), expressed as percentage of control condition. Bar charts represent the average ± SEM for control (*n* = 7) and h‐iAβo (*n* = 6) cells. * denotes *p* < 0.05

Previous evidence indicates that Aβ disrupts excitatory neurotransmission in different AD mice models (Bell et al., 2007; Hascup et al., 2019; Hascup & Hascup, 2015; Šišková et al., 2014) thereby affecting E/I balance (Lei et al., 2016). Since our previous results showed an increase in iAβo‐modulated neurotransmission and augmented spike number in current recording mode, we examined whether h‐iAβo could specifically affect glutamatergic vs GABAergic neurotransmission thereby changing the E/I balance in hippocampal neurons. For this, we performed voltage‐clamp experiments using a low Cl^−^ internal solution containing QX‐314 (see details in Section 2.4.1). This allowed to separately record EPSC and IPSC in a single cultured neuron by changing the membrane holding potential and in the absence of spikes at positive potentials. The data showed that h‐iAβo markedly increased the spontaneous excitatory post‐synaptic currents (sEPSC) (Figure 3a, bottom, right trace). The data also show that inhibitory post‐synaptic currents (sIPSC) were affected to a lower extent in this experiment, possibly because the sIPCS frequency in control conditions was already high. The data also show that the charge transferred for sEPSC, but not sIPSC, was significantly increased by h‐iAβo (Figure 3b, black bar). Consequently, under these experimental conditions, the E/I balance obtained analyzing charge transferred was highly and rapidly increased by h‐iAβo (Figure 3b, white bar). Since sEPSC are mainly mediated by AMPA receptors in the current experimental conditions (−60 mV, external Mg^2+^), our results suggest that the alteration in AMPA‐mediated neurotransmission underlies this excitatory input disruption.

### 2.4 **Effects of iAβo on isolated excitatory AMPA and inhibitory GABA_A_ miniature and evoked currents**.

**FIGURE 4:**
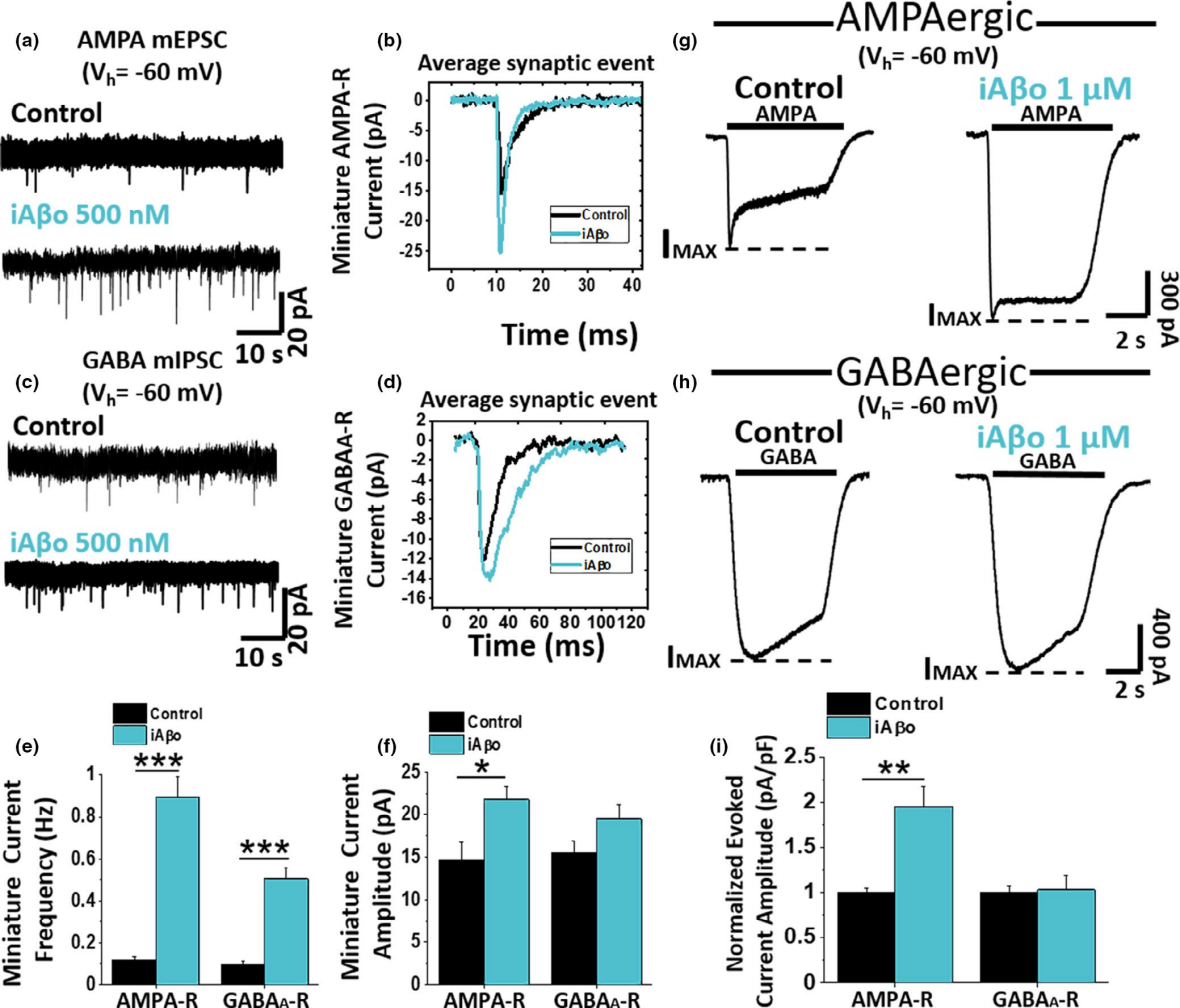
iAβo differentially affected AMPA‐R and GABA_A_‐R mediated miniature and evoked PSC *in vitro*. (a– d) Pharmacologically isolated AMPA (a) and GABA (c) mPSC in control condition and with 500 nM iAβo at *V*_h_ = −60 mV. AMPA miniature currents (mEPSC) were isolated perfusing (in µM): 20 DAPV, 1 strychnine, and 10 bicuculline, while GABA miniature currents (mIPSC) were isolated using (in µM): 20 DAPV, 1 strychnine, and 20 CNQX. The recordings show the averaged synaptic current for each condition (b and d). e, f, mEPSC (*n* = 9) and mIPSC (*n* = 8) frequency (e) and amplitude quantification (f) for control and iAβo‐treated cells (see Figure S4 for *ex vivo* results and Figure S5 for AMPA‐R mEPSC obtained in the presence of h‐iAβo). (g, h) AMPA (g) and GABA (h) evoked currents in the absence (*n* = 9) and the presence of 1 µM iAβo (*n* = 9). 100 µM GABA and AMPA were perfused to evoke maximal amplitude currents in the presence of 500 nM TTX. Segmented lines indicate maximum current reached (*I*_MAX_). i, Normalized evoked current amplitude (value corresponding to I_MAX_) for AMPA and GABA receptors. Bar charts represent the average ± SEM. Unpaired Student's *t* test with Welch's correction for mEPSC frequency: *t*(8.29) = −8.07, *p* = 3.31E‐5 and unpaired Student's *t* test for mIPSC frequency: *t*(14) = −7.43, *p* = 3.20E‐6. Unpaired Student's *t* test for mEPSC amplitude: *t*(16) = −2.62, *p* = 0.018 and mIPSC amplitude: *t*(14) = −1.86, *p* = 0.086. Unpaired Student's *t* test with Welch's correction for (i) AMPA‐R: *t*(16) = −3.83, *p* = 1.4E‐3 and GABA_A_‐R: *t*(16) = 0.17, *p* = 0.863. * denotes *p* < 0.05, ** *p* < 0.01 and *** *p* < 0.001

Next, we examined pharmacologically isolated AMPAergic and GABAergic miniature synaptic currents [two of the predominant neurotransmissions present in cultured hippocampal neurons (Vizi & Kiss, 1998)] to determine the presence of selectivity of iAβo on both neurotransmissions. iAβo caused a significant increase in the frequency of both AMPA mEPSC and GABAergic mIPSC (Figure 4a,c). On the other hand, only the amplitude of AMPA was affected by iAβo, as seen by the average synaptic event (Figure 4b). The quantification of these parameters (frequency and amplitude) showed that iAβo affects both types of neurotransmissions, with the largest increases in both AMPA parameters (Figure 4e,f). Additional experiments in the stratum pyramidale of the CA1 area (SP‐CA1) in hippocampal slices of an adult mouse brain (*ex vivo*) showed similar results and support the idea of a larger effect of iAβo on excitatory currents (Figure S5). Because the AMPAergic‐mediated neurotransmission was found to be more significantly affected than the GABAergic (in terms of frequency and amplitude), we decided to confirm this result by examining AMPAergic currents in the presence of h‐iAβo and also found an increase in neurotransmission (Figure S6). The result showing that the amplitude of AMPA synaptic currents was affected with human‐derived and synthetic preparations was also confirmed evaluating ligand‐evoked currents in cultured hippocampal neurons. The data showed that the presence of iAβo enhanced the AMPA‐mediated current amplitude by about two times (Control I normalized: 1.00 ± 0.05 vs. iAβo: 1.95 ± 0.23) (Figure 4g,i). On the contrary, no effects were observed for GABA‐evoked currents (Figure 4h,i).

### 2.5 The effect of iAβo on AMPA neurotransmission depends on PKC

**FIGURE 5:**
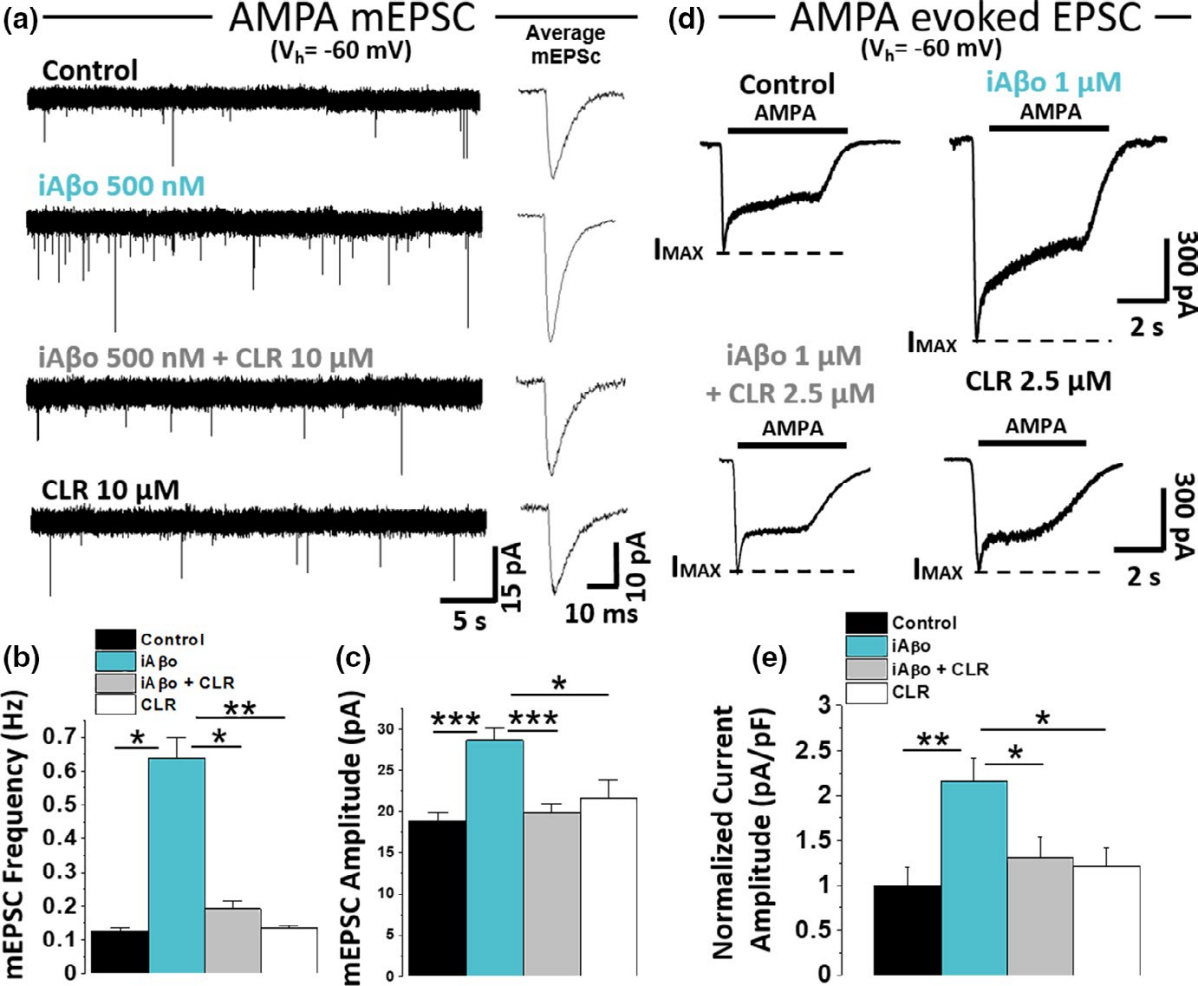
The effect of iAβo on miniature AMPAergic and evoked EPSC is PKC‐dependent. (a) mEPSC recordings of primary hippocampal neurons obtained for control (*n* = 11), 500 nM iAβo (*n* = 11), 2.5 µM chelerythrine (CLR) +500 nM iAβo (*n* = 12), and 2.5 µM CLR (*n* = 12) (*V*_h_ = −60 mV). AMPA miniature currents (mEPSC) were isolated perfusing (in µM): 20 DAPV, 1 strychnine, and 10 bicuculline, in the presence of 500 nM TTX and recorded at *V*_h_ = −60 mV. (b, c) mEPSC frequency (b) and amplitude quantification (c) for each of the conditions described in (a). (d) AMPA‐evoked current in the absence (*n* = 9) and the presence of 1000 nM iAβo (*n* = 9), iAβo co‐applied together with CLR (*n* = 11) and CLR (*n* = 11) (V_h_ = −60 mV). 100 µM AMPA was perfused to evoke maximal amplitude currents in the presence of 500 nM TTX. Segmented lines indicate maximum current reached (*I*_MAX_). (e) Average amplitude of AMPA‐evoked current for each condition in (d). Bar charts represent the average ± SEM. One‐Way Welch's ANOVA with Games–Howell *post hoc* test for (b): *F*(3, 42) = 6.11, *p* = 0.002. p‐values for *post hoc* test: control vs. iAβo: 2.27E‐2, iAβo vs. iAβo + CLR: 1.78E‐2, iAβo vs. CLR: 2.07E‐3. One‐Way Welch's ANOVA with Games–Howell *post hoc* test for (c): *F*(3, 42) = 8.54, *p* = 1.52E‐4. P‐values for *post hoc* test: control vs. iAβo: 2.65E‐4, iAβo vs. CLR: 1.23E‐2, iAβo vs. iAβo + CLR: 9.49E‐4. One‐Way Welch's ANOVA with Games–Howell *post hoc* test for (e): *F*(3, 35) = 5.88, *p* = 2.31E‐3. p‐values for *post hoc* test: control vs. iAβo: 3.31E‐3, iAβo vs. CLR: 1.10E‐2, iAβo vs. iAβo + CLR: 1.49E‐2. * denotes *p* < 0.05, ** *p* < 0.01 and *** *p* < 0.001

These previous results prompted us to investigate in depth the mechanism(s) that could be producing the effect of iAβo on the AMPAergic excitatory transmission. Phosphorylation of ionotropic channels plays a preponderant role in the regulation of synaptic function (Raymond et al., 1993), and the intracellular perfusion of an atypical isoform of PKC denominated PKCM or PKM (a constitutively active form of PKC) increases the synaptic response mediated by the AMPA receptor in a similar fashion to that induced by iAβo (Ling et al., 2006). Therefore, we examined miniature (left, Figure 5a) and ligand‐evoked (right, Figure 5d) currents in cultured neurons in the presence of a PKC inhibitor (CLR, 2.5 µM intracellularly) finding that the effect of iAβo on the frequency and amplitude of the mEPSCs was reduced when co‐applied with CLR (Figure 5b,c). These results clearly show that the effect of iAβo on AMPA mEPSCs was dependent on this kinase. The effect of iAβo on the current evoked by AMPA was also reduced by the PKC inhibitor (Figure 5d,e). These results were interesting because it appears that PKC is actually linked to the effect of iAβo on AMPA‐mediated responses (a post‐synaptic effect) and on the frequency of mEPSC (pre‐synaptic effect), suggesting that the global effects of iAβo on the excitatory synapse through this kinase compromise the pre‐ and post‐synaptic functions.

### 2.6 iAβo increases NO production in the recorded (RN) and neighboring (NN) neurons

**FIGURE 6:**
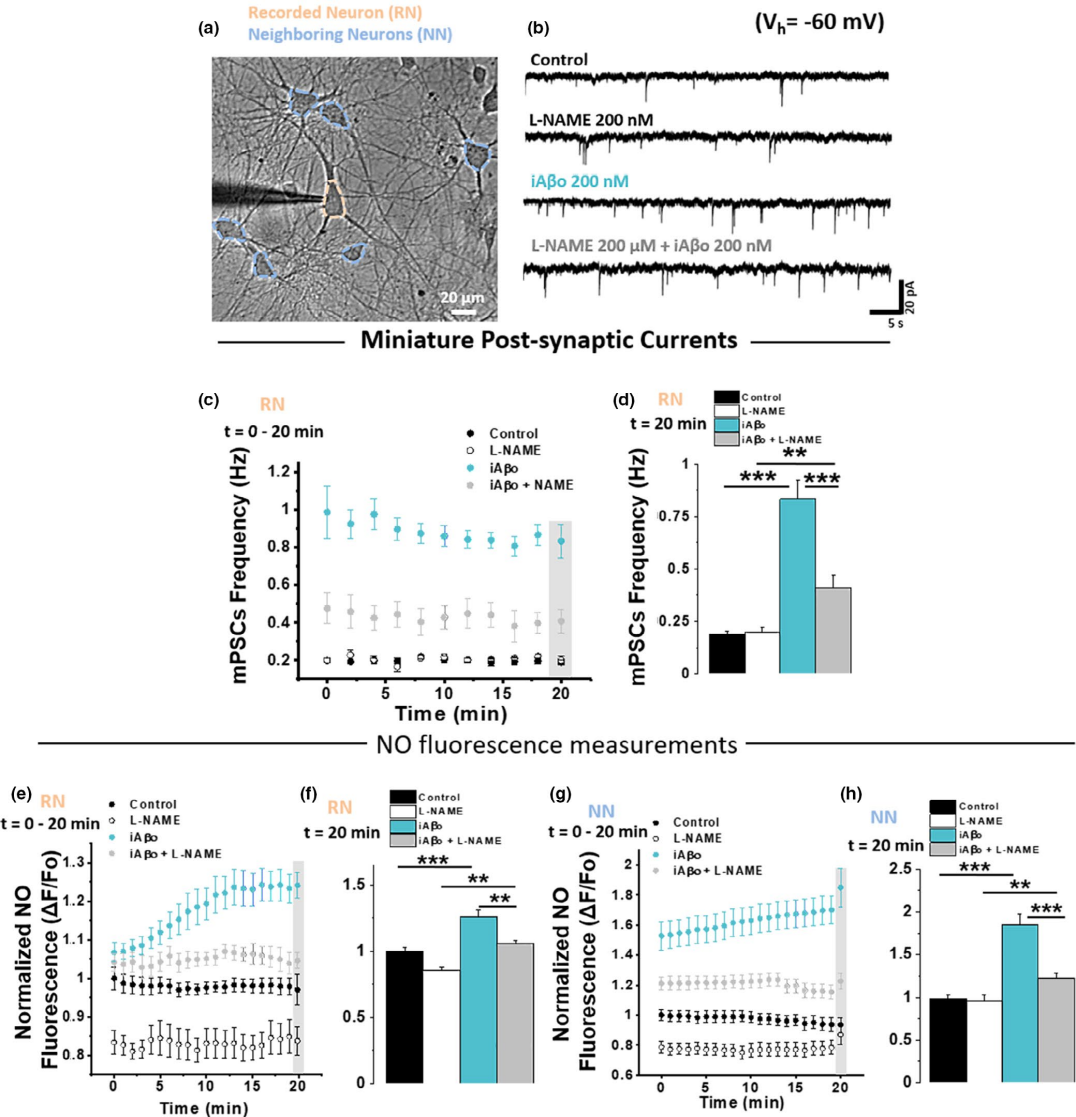
Nitric oxide synthase is important for the effects of iAβo on the frequency of miniature synaptic currents in the neuronal ensemble. (a) Micrograph showing the area of the primary hippocampal culture in which mPSC were measured from the recorded neuron (RN), as well as the NO fluorescence for RN and of the neighboring neurons to RN (NN). (b, c) Representative recordings (b) and quantification of mPSC frequency (c) obtained during the course of 20 min in the absence (control) and the presence of 200 nM iAβo, together with the pre‐incubation with L‐NAME (200 µM for 20–30 min). mPSC were recorded in the presence of 500 nM TTX (*V*_h_ = −60 mV). (d) Quantification of mPSC frequency at 20 min (obtained from data marked inside gray rectangle from plot in (c). (e–h) Relative levels of NO (expressed as normalized fluorescence) obtained throughout the course of the experiment and at 20’ for RN (e and f, respectively) and NN (g and h, respectively). See details in Section 2.6. Line and bar graphs represent the average ± SEM. Control (*n* = 9), L‐NAME (*n* = 8), iAβo (*n* = 11) and iAβo + L‐NAME (*n* = 10) for RN and control (*n* = 97), L‐NAME (*n* = 102), iAβo (*n* = 119) and iAβo + L‐NAME (*n* = 102) for NN. One‐Way Welch's ANOVA with Games–Howell *post hoc* test for (d): *F*(3, 34) = 60.309, *p* = 1.07E‐13. p‐values for *post hoc* test: Control vs. iAβo: 1.06E‐08, L‐NAME vs. iAβo + L‐NAME: 7.80E‐3, iAβo vs. iAβo + L‐NAME: 1.40E‐08. One‐Way Welch's ANOVA with Games–Howell *post hoc* test for (f): *F*(3, 34) = 21.926, *p* = 4.41E‐8. p‐values for *post hoc* test: Control vs. iAβo: 2.53E‐05, L‐NAME vs. iAβo + L‐NAME: 3.03E‐3, iAβo vs. iAβo + L‐NAME: 1.18E‐03. One‐Way Welch's ANOVA with Games–Howell *post hoc* test for (h): *F*(3, 416) = 40.763, *p* = 4.11E‐23. p‐values for *post hoc* test: Control vs. iAβo: 6.44E‐20, L‐NAME vs. iAβo + L‐NAME: 2.16E‐3, iAβo vs. iAβo + L‐NAME: 8.85E‐09. ** denotes *p* < 0.01, *** *p* < 0.001. See Figure S6 for experiments obtained in the presence of SNAP and CPTIO and Figure S7 for experiments using iNOS inhibitor (1400W)

The data showed that there was a potent effect on the frequency of mPSC when iAβo was dialyzed into the post‐synaptic neuron, suggesting an increase in the release of neurotransmitters at the pre‐synaptic terminal (Malgaroli & Tsien, 1992). Could it be that iAβo applied to the post‐synaptic neuron affects the release of neurotransmitters occurring at the pre‐synaptic neuron? If so, could there be a retrograde mechanism involved in the effect? (Suvarna et al., 2016). Because the effect was rapid, we thought that it could be produced by nitric oxide (NO), a retrograde messenger, known to be involved in pre‐synaptic neurotransmitter release (Arancio et al., 1996; Hardingham et al., 2013; Hawkins et al., 1998) and produced by nitric oxide synthase (NOS). It is important to note that 200 nM of iAβo was used for all the recordings obtained in these experiments (in the presence of TTX), which did not affect mPSC amplitude, but only its frequency. First, we used a non‐selective nitric oxide synthase inhibitor (L‐NAME) and monitored mPSC frequency with electrophysiology and relative NO levels with a fluorescent probe (DAQ) (details in methods Section 2.6). This allowed us to monitor relative levels of NO in the recorded cell (denoted as RN) (Figure 6a) and in the neighboring neurons (denoted as NN) (Figure 6a). Pre‐incubation of hippocampal neurons with L‐NAME showed no significant effects per se in the frequency of miniature currents (Figure 6b–d), but it did significantly decrease the NO levels in RN (Figure 6e) and in NN (Figure 6g) when compared to control conditions for RN and NN, respectively. The statistical comparison was performed at the end of the recording period (20 min) (Figure 6e,g, indicated by the gray box) and plotted as a bar graph for RN (Figure 6f) and NN (Figure 6h). For its part, iAβo had the expected effect and increased the frequency of mPSC (Figure 6b–d). Interestingly, the increase in iAβo‐mediated miniature synaptic activity occurred at the same time that NO levels increased in the RN (Figure 6e,f). More importantly, iAβo not only caused a fast increase in the levels of NO in the RN but also in NNs, not dialyzed with iAβo (Figure 6g,h). Pre‐incubation with L‐NAME reduced the effect of iAβo on mPSC frequency by ≈ 51% (Figure 6b–d) in the RN. This decrease in the frequency of mPSC correlated well with the decrease in the relative NO levels in the RN and NN (Figure 6e–h, iAβo + L‐NAME). Second, a NO‐dependent effect was confirmed by co‐applying iAβo together with a donor molecule for NO (SNAP 300 µM) causing a synergistic effect and increasing the frequency of mPSC in the RN (Figure S7), as well as in the relative levels of NO in both the RN and NN (Figure S7). The opposite effect was observed using iAβo in the presence of the NO chelator CPTIO (Figure S7). On the other hand, no changes in the effects of iAβo in the frequency of mPSC were found using a specific inhibitor of inducible NO (iNOS) (Figure S8).

### 2.7 iAβo increased neuronal excitability of hippocampal neurons *in vivo* and *in vitro*

**FIGURE 7:**
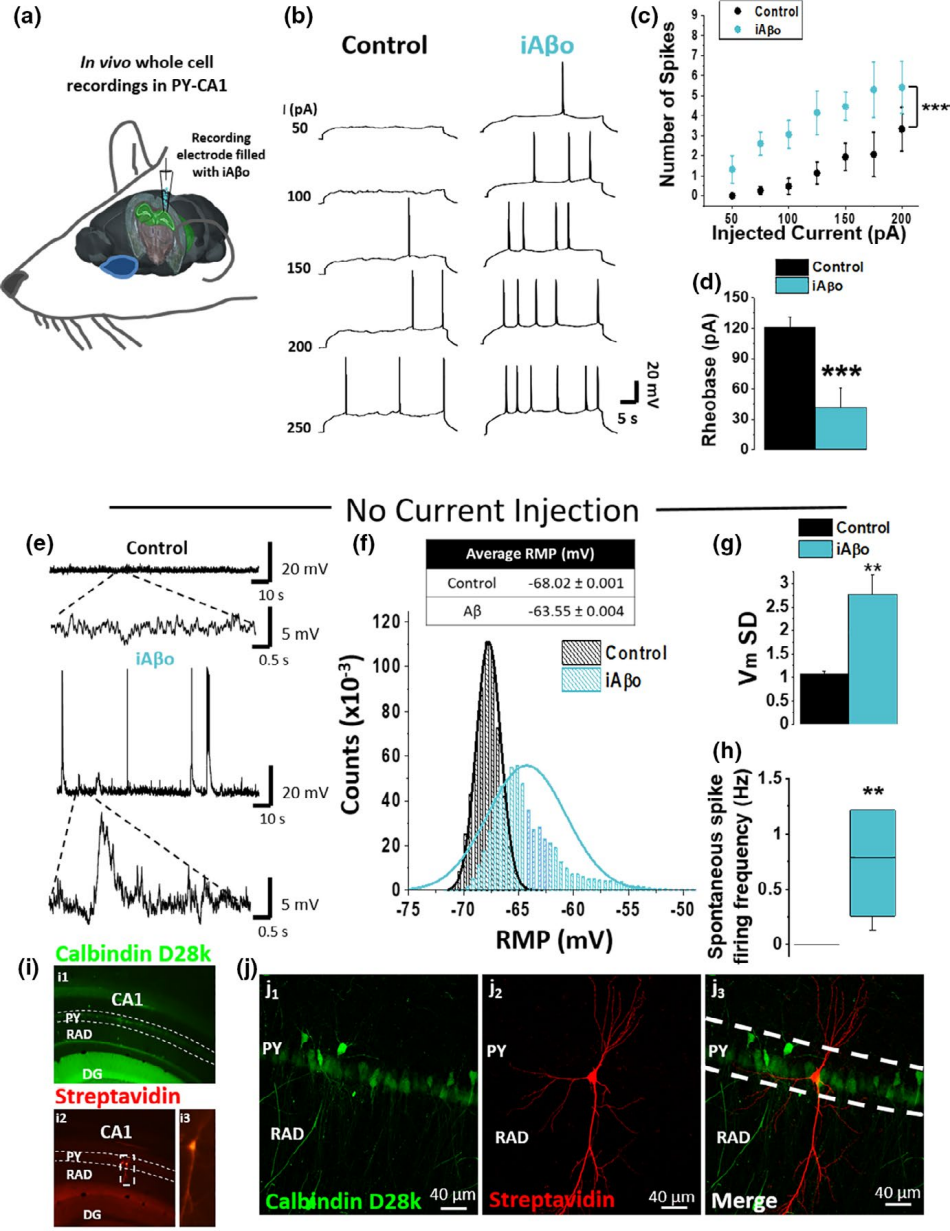
iAβo increased the firing of action potentials evoked by current injection in hippocampal neurons *in vivo*. (a) Scheme representing the location of the electrode filled with iAβo to record action potentials in the pyramidal cell layer of CA1 (PY) of rat hippocampus (highlighted in green) (brain image obtained from Allen Institute Brain Atlas). (b) Representative action potential recordings in the absence and the presence of 500 nM iAβo obtained in current‐clamp mode (details in Section 2.4.3). (c) Relationship between the number of evoked action potentials and the injected current intensity for the experimental conditions described in (b) (two‐way ANOVA: *F*(7, 103) = 7.983, *p* = 1.01E‐7). (d) Quantification of the rheobase constant (unpaired Student's *t* test: *t*(14) = 4.188, *p* = 9.11E‐4). For Rin and kinetic parameters of APs, see Figure S8. e, Representative recordings obtained without injection of depolarizing current pulses after stabilizing the resting membrane potential (RMP) to −70 mV. (f) *V*_m_ values histogram and average values of *V*_m_ ± SEM. (g, h) Quantification of standard deviation (SD) values for *V*_m_ (g) (unpaired Student's *t* test: *t*(14) = −3.445, *p* = 3.94E‐3) and spontaneous AP firing frequency (h) (Mann–Whitney *U* test: U=0, z‐score=−3.199, *p* = 1.38E‐3). (i) Epifluorescence micrographs of coronal cuts obtained after the electrophysiological recording showing a positive staining for calbindin‐D‐28k (in green, i_1_) and streptavidin (in red, i_2_) in hippocampus‐CA1. The recorded neuron (i_3_) is also observed. DG, dentate gyrus; PY, pyramidal cell layer of CA1; RAD, stratum radiatum. (j) Confocal micrographs demonstrating the positive staining for streptavidin in the recorded neuron (in red, j_2_), as well as the merge with calbindina‐D‐28k (j_3_) within the stratum pyramidale of the CA1 region of the hippocampus. Bar and line charts represent the average ± SEM for control (*n* = 10) and h‐iAβo (*n* = 6) cells of at least six rats. ** denotes *p* < 0.01, *** *p* < 0.001

The previous results showed that the excitatory transmission and E/I balance are strongly affected by human and synthetic iAβo in cultured neurons. The CA1 area in the hippocampus (a major center of excitatory neurotransmission) is one of the brain regions prone to develop early AD neuropathology in humans and mice models (Braak et al., 2006; Gouras et al., 2000; Kerchner et al., 2010; LaFerla et al., 2007; Padurariu et al., 2012); therefore, we studied hippocampal CA1 pyramidal neurons using whole‐cell current‐clamp recordings in an anesthetized *in vivo* rat model to examine neuronal excitability (Figure 7a). We found that in the presence of iAβo, the neuron began spiking in response to lower current stimuli (Figure 7b). Plotting the number of spikes vs. injected current showed a strong shift of the curve to the left (Figure 7c), implying that the cell with iAβo was now more excitable. Indeed, the constant rheobase for firing diminished in neurons treated with iAβo (Figure 7d). On the other hand, the calculated values for input resistance (*R*_in_) and kinetic parameters of action potentials (APs) (amplitude, duration, and threshold) in both conditions were similar to control conditions (Figure S9). Furthermore, recording of resting membrane potentials (*V*_m_), without current injection, showed a large increase in *V*_m_ fluctuations when iAβo was applied to the neuron (Figure 7e). The large fluctuations of *V*_m_ in neurons treated with iAβo appear to result from the synaptic potentials (Figure 7e, lower trace), some depolarizing the cell up to the threshold for AP firing (Control: −68.02 ± 0.001 v/s iAβo: −63.55 ± 0.004; Figure 7f). This was also evidenced as a significant change in the standard deviation (SD) of *V*_m_ (Figure 7g). iAβo also increased the chance of spontaneous spike firing (Figure 7h) (control: 0.003 ± 0.003 Hz vs. iAβo: 1.068 ± 0.463 Hz). Post‐recording immunohistochemical analysis confirmed the location of the recorded neurons to be in the stratum pyramidale of the dorsal hippocampus at CA1 (Figure 7i,j).

**FIGURE 8:**
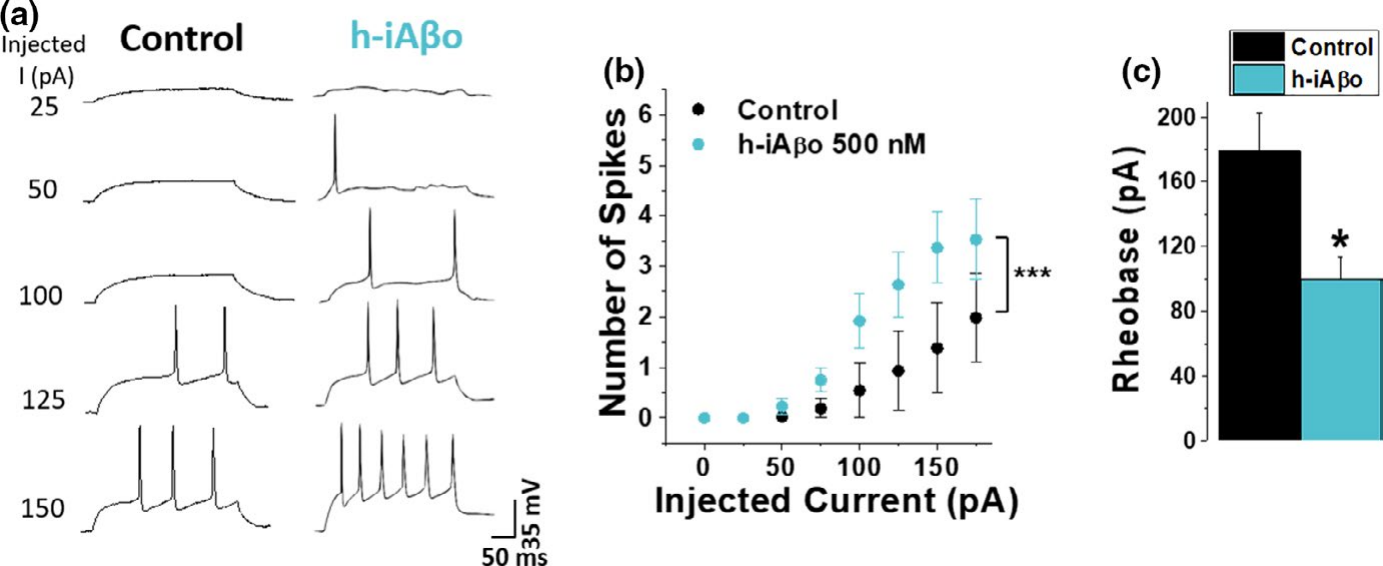
Intracellular human‐derived Aβ oligomers (h‐iAβo) increased the firing of action potentials in hippocampal neurons *in vitro*. (a) Action potential (AP) firing in primary hippocampal neurons in the absence and the presence of 500 nM h‐iAβo obtained in current‐clamp mode (details in Section 2.4.3). (b) Relationship between the number of AP spikes and the injected current intensity for the experimental conditions described previously (two‐way ANOVA: *F*(8, 79) = 9.077, *p* = 9.31E‐9). (c) Rheobase constant decreased ≈45% for h‐iAβo condition (unpaired Student's *t* test: *t*(9) = 2.737, *p* = 2.29E‐2). Bars and line charts represent the average ± SEM for control (*n* = 6) and h‐iAβo (*n* = 5) cells. * denotes *p* < 0.05, *** *p* < 0.001. For results obtained with synthetic iAβo, see Figure S9

After determining the effects of iAβo on neuronal excitability in an *in vivo* model, and with the aim of examining the effect of human‐derived iAβo that is relevant for the human disease, we studied neuronal excitability in cultured hippocampal neurons. In agreement with the previous data (Figure 7), we found that a smaller current injection was required to trigger the firing of APs in the presence of h‐iAβo (Figure 8a). Plotting the number of spikes vs. injected current demonstrated that the stimulus‐response curve shifted to the left indicating an increase in neuronal excitability in the presence of h‐iAβo (Figure 8b) together with a reduced rheobase (Figure 8c). Similar results were obtained for synthetic iAβo in the cultured neuron model (Figure S10), confirming the effect of human and synthetic preparations on neuronal excitability *in vitro*.

**FIGURE 9:**
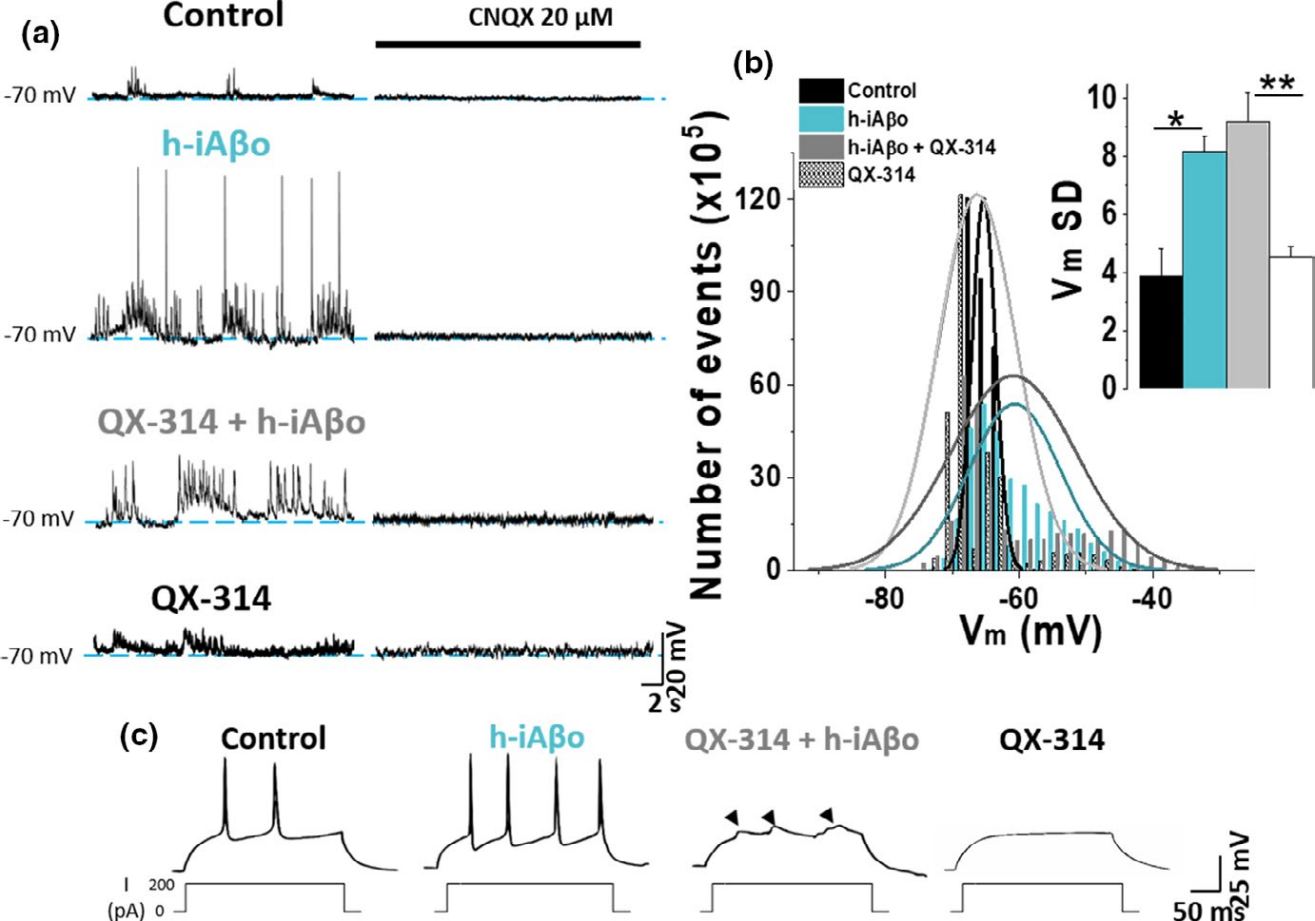
Intracellular blockade of voltage‐regulated Na_v_ channels does not prevent depolarization of the membrane activated by h‐iAβo *in vitro*. (a) Representative recordings obtained without current injection showing membrane potential (*V*_m_) fluctuations under the different conditions tested. External application of 20 µM CNQX through a perfusion line inhibited the fluctuations to a great extent in all conditions. (b) Histogram showing the distribution of *V*_m_ values in the different experimental conditions shown in (a), along with the *V*_m_ SD (inset bar graph) One‐Way Welch's ANOVA with Games–Howell *post hoc* test for *F*(3, 19) = 11.299, *p* = 1.77E‐4. p‐values for *post hoc* test: Control vs. iAβo: 7.13E‐3 and QX‐314 vs. QX‐314 + iAβo: 2.12E‐3). (c) Current injection experiments demonstrate that, under the control and h‐iAβo conditions, the generation of action potentials was not inhibited, while Na_v_ intracellularly blocked by 5 mM QX‐314 prevented spiking of neurons with h‐iAβo and without it. Arrowheads over the third trace (left‐to‐right direction) indicate the presence of depolarizing post‐synaptic potentials when h‐iAβo was present. This did not occur for the condition with QX‐314 alone (4th trace; left‐to‐right direction). Bars represent the average ± SEM for control (*n* = 6), h‐iAβo (*n* = 5), h‐iAβo+QX‐314 (*n* = 6) and QX‐314 (*n* = 6) cells. * denotes *p* < 0.05, ** *p* < 0.01 and *** *p* < 0.001. For results obtained with synthetic iAβo, see Figure S10

We then evaluated whether the effect of h‐iAβo on the increase in APs firing could be the result of augmented transitory depolarizations at the post‐synaptic level due to the increase in AMPAergic neurotransmission. In order to examine this, we applied h‐iAβo and recorded the membrane potential (*V*_m_) fluctuations of cultured hippocampal neurons without current injection in the presence and the absence of an intracellular voltage‐dependent sodium channel (Na_v_) blocker (5 mM QX‐314). When Na_v_ was blocked (Figure 9a, last trace), APs were not activated in the recorded neuron, as expected. In control conditions, *V*_m_ fluctuations generated a population of local synaptic potentials (Figure 9a) with an average *V*_m_ value of −65.3 ± 0.003 mV (Figure 9b). In the presence of h‐iAβo, there was a significant increase in the number and amplitude of these depolarizing events (Figure 9a), with an average of −60.6 ± 0.012 mV (Figure 9b), similar to what we observed previously in the *in vivo* model. In the presence of iAβo and QX‐314, no major differences were observed in *V*_m_ fluctuations with respect to h‐iAβo, except for the lack of AP firing (Figure 9a). The data show a similar average *V*_m_ value to the one observed when h‐iAβo was present (−60.8 ± 0.017 mV) (Figure 9b). On the other hand, *V*_m_ in the presence of QX‐314 had a similar average value to control condition with an average of −65.1 ± 0.014 mV (Figure 9a‐b, in gray). Similar results were obtained in the presence of synthetic iAβo (Figure S11). It is interesting to note that in all tested conditions, the external application of an AMPA receptor antagonist (CNQX) through the perfusion system attenuated all *V*_m_ fluctuations (Figure 9a), indicating that these changes in membrane potential in our experimental condition were synaptic AMPA‐driven potentials. This confirms that the AMPAergic‐mediated synaptic input is responsible for the iAβo‐mediated hyperexcitability observed in the experiments. Additionally, in the presence of intracellular QX‐314, no spikes (APs) were detected (Figures 9c, 3^rd^ and 4^th^ traces; left‐to‐right direction) confirming blockade of Na^+^ channels. We also observed the presence of transitory depolarization (Figure 9c, arrowheads over 3^rd^ trace; from left to right) that were not found in the presence of QX‐314 alone (Figures 9c, 4^th^ trace; from left to right). These data indicate that iAβo exerts depolarizations of the post‐synaptic membrane even in the absence of APs, suggesting that this effect did not depend on the generation of APs at the post‐synaptic level, but rather an increase in AMPA synaptic transmission at pre‐ and post‐synaptic levels.

## 3 DISCUSSION

A significant number of studies have shown the presence of Aβo in the intracellular compartment, including organelles such as mitochondria, endoplasmic reticulum, Golgi network, lysosomes (reviewed in (LaFerla et al., 2007). Intracellular Aβ accumulation has also been reported in the hippocampus of healthy subjects [regardless of gender or age (Blair et al., 2014)], but an important difference from AD brains is that in the latter these accumulations constitute oligomeric forms of Aβ. Additional evidence suggests that soluble oligomeric Aβ directly inhibits the proteasome *in vivo* (Tseng et al., 2008). This intracellular accumulation is pathological since it leads to the accumulation of tau protein (Tseng et al., 2008). Since the proteasome is found in the cytosolic fraction, cytosolic Aβ must be at a considerable quantity to exert these pathogenic roles. Indeed, its cytosolic presence has also been suggested by other authors (Bückig et al., 2002; Lee et al., 2006; Zheng et al., 2012, 2013). Therefore, we wanted to examine whether the application of Aβo to the intracellular milieu was able to alter some properties of hippocampal neurons to link the presence of intracellular Aβ to functional results. The results showed that the presence of intracellular oligomeric species of Aβ increases neurotransmission through mechanisms that involved pre‐and post‐synaptic actions that resulted in an increased excitability and spreading of neuronal activation. Because the present findings were obtained with human AD brain‐derived and synthetic Aβo, they should contribute with disease‐relevant data in a new and significant working model. Although the effects of both the human‐derived peptide and the synthetic peptide were extensively similar, the present study has an important constraint in that we were not able to fully biochemically characterize the oligomeric preparations and determine the yields of each species present. In general, they do not appear to be monomers or fibers, to a large extent. From previous studies, employing biochemical, transmission electronic microscopy, and AFM analysis (González‐Sanmiguel et al., 2020; Peters et al., 2016, 2020), we concluded that most of Aβ are in its soluble oligomeric form, from dimers to small protofibrils. More importantly, we showed that the acute application of these resulting Aβ species, at low concentrations (0.5–1 μM), dissolved in physiological solution similar to the present study, produced increases in intracellular calcium and synaptic transmission in hippocampal neurons allowing us to conclude that these species are diffusible and interact with cell membranes. Despite these limitations, we believe that our results are consistent to support an excitatory effect of intracellular Aβ, thus validating the importance of the intracellular oligomeric species of Aβ in the disease. In addition, supporting the notion that iAβ affects physiological neuronal parameters, we recently reported that accumbal neurons in the APP/PS1 Tg AD mice displayed an increase in neuronal excitability in the presence of intracellular, but not extracellular Aβ (Fernández‐Pérez et al., 2020). Therefore, the use of these preparations in *in vitro*, *ex vivo*, and *in vivo* models allowed us to mechanistically understand the effects on neurotransmission and excitability that could be important in the initial stages of AD and that is likely to play a major role in an AD brain.

The question whether AD is accompanied by increases in neuronal excitability and clinically expressed as seizures during disease progression remains under debate (Hauser et al., 1986; Hesdorffer et al., 1996; Sjogren et al., 1953). However, a number of studies have supported this possibility (Brorson et al., 1995; Chen, 2005; Good & Murphy, 1996; Mark et al., 1995; Mattson et al., 1992). In mice models, an increase in excitability was reported in different brain regions such as the cortex (Kellner et al., 2014), nucleus accumbens (Fernández‐Pérez et al., 2020), thalamus (Gurevicius et al., 2013), and hippocampus (Bezzina et al., 2015; Born et al., 2014; Busche et al., 2012; Davis et al., 2014; Del Vecchio et al., 2004; Minkeviciene et al., 2009). Neuronal excitability is mainly dependent on passive (i.e., resting potential, input resistance) and active (ion channels) membrane properties that control the ability of the neuron to fire APs. Thus, it was previously shown that intracellular Aβ increases neuronal excitability by the suppression of BK (Yamamoto et al., 2011) or A‐type Kv channels (Scala et al., 2015). In this study, we found that human and synthetic iAβo increased excitatory neurotransmission and reduced rheobase leading to an increase in excitability in the absence of changes in AP properties. Therefore, this study provides relevant experimental evidence and a novel intracellular mechanism to explain the hyperexcitability observed early in the disease that might finally lead to neurodegeneration.

Other previous studies have examined the effects of intracellular Aβo on neurotransmission. For example, it was shown that 200 pM intracellular Aβo was unable to affect long‐term potentiation (LTP); however, extracellular Aβo increased LTP (Gulisano et al., 2019). Unlike the present study, no effects were produced on AMPA‐evoked currents when the peptide was applied at 200 pM (either internally or externally) and the changes were attributed to alterations in nAchRα7 (Gulisano et al., 2019). Another study found a reduction in mEPSC after 20 min of dialyzing 200 nM intracellular Aβo using hippocampal autaptic synapsis (Ripoli et al., 2014). The latter study supports the need for a more complex synaptic circuitry that can only be obtained in well‐connected neurons and mature brain slices like the ones used in the present study. In addition, the rapid effect that we found supports the notion that low concentrations of iAβo are needed to affect these synapses. Considering that the molecular weight of Aβ is above 4500 Da and that the time of equilibration with the patch pipettes used (Raccess ~10 MOhms) (Pusch & Neher, 1988) should be on the order of 10–20 min, the increase in transmission already at 1 min supports the idea that low concentrations of iAβo are needed to affect the synapsis. The data with fluorescent Aβ entry to the cell support the notion of a rapid entry and subsequent effect of iAβo. Interestingly, the dialyzed iAβo was able to affect other neighboring neurons as detected using a NO probe.

This raises the question of how iAβo actions could progress to synaptic dysfunction in the first place? In this regard, it has been reported that extracellular Aβo increases glutamate neurotransmission (Parodi et al., 2010) which could lead to excitotoxicity (Harkany et al., 2000; Mehta et al., 2013; Olloquequi et al., 2018) together with vesicular depletion, decreased phosphorylation, and localization of the AMPA and NMDA receptor in synapses when Aβ is present in the extracellular milieu over extended periods of time [for review see (Crews & Masliah, 2010; Marsh & Alifragis, 2018)]. Considering the present results, it is tempting to speculate that iAβo might be contributing to an important release of excitatory neurotransmitters [such as glutamate and D‐serine (Paula‐Lima et al., 2013)] and post‐synaptic current potentiation, but over time, iAβo influences synaptic vesicle depletion as well as reduced post‐synaptic receptor phosphorylation and localization, contributing to synaptic failure. This is in agreement with results from murine AD models that show that intracellular accumulation of Aβo leads to synaptic failure and the onset of cognitive deficits [for further details see (Bayer & Wirths, 2010)], again emphasizing the importance of iAβo during the pre‐symptomatic stage of AD.

### 3.1 Pre‐synaptic mechanism: Increase in retrograde synaptic signaling by NO

Simultaneous fluorescence and electrophysiology recordings demonstrated that NO is involved in the pre‐synaptic effect of iAβo. The data showed that iAβo applied to the post‐synaptic site augmented NO levels globally together with the frequency of mPSC, reflecting an enhanced release of neurotransmitters into the synaptic space (Malgaroli & Tsien, 1992), which is in agreement with studies that have shown that NO increases neurotransmitter release from excitatory and inhibitory synapses (McNaught & Brown, 2002; Prast & Philippu, 2001; Zanelli et al., 2009). Furthermore, PKC is likely involved in this pre‐synaptic effect of iAβo since CLR prevented the iAβo‐mediated increase in the mEPSC frequency. In agreement with this notion, it was reported that PKC also participates in pre‐synaptic mechanisms mediated by the gas neuromodulator NO (Leppänen et al., 2014; Ping et al., 1999; Song et al., 2008), emphasizing the pathway that iAβo might be using to increase neurotransmission.

The idea that NO signaling is involved in the iAβo‐mediated alterations is supported by the increase of this messenger in an ensemble of neurons that were near the one dialyzed. Also, during the development of AD, NO synthases (NOS) were found to increase in human brains and rodent models (Lüth et al., 2002; Shilling et al., 2014). Moreover, in pre‐symptomatic 3xTg mice, alterations that favor NO‐synthesis and occur together with synaptic pathology have been reported (Shilling et al., 2014). Finally, at the cellular level a prolonged release of NO could cause metabolic, oxidative stress, and dysfunction in organelles such as mitochondria and ER, eventually leading to neuronal death (Andersen, 2004; Moncada & Bolaños, 2006; Moncada & Higgs, 2006; Sayre et al., 2008; Steinert et al., 2010). Thus, we can hypothesize that unregulated release of NO in AD pathology might have negative consequences for neuronal function beginning at the synapsis. Therefore, the results are in agreement with other studies indicating that NO signaling is altered in AD.

### 3.2 Post‐synaptic mechanism for AMPA transmission potentiation by iAβo

The data indicate that iAβo caused a post‐synaptic potentiation through the increase in AMPA‐mediated currents. Changes in mEPSC amplitude mostly reflect alterations at the post‐synaptic level (Manabe et al., 1992). However, changes might also be explained by an increased neurotransmitter content in synaptic vesicles, translating into an increase in the amplitude of post‐synaptic currents (Kullmann & Nicoll, 1992). This does not appear to be the case for iAβo because the direct activation of AMPA receptors, using a saturating concentration of the agonist, still increased the amplitude of the current, supporting the idea that the effect was post‐synaptic in nature. The amplitude of AMPA‐evoked and miniature AMPAergic currents (mEPSC) increased in the presence of iAβo, an effect that was largely attenuated by a PKC inhibitor. Interestingly, alterations in PKC activity have also been reported in AD. For example, an impaired function of PKC was reported in human *postmortem* AD brains (Cole et al., 1988; Wang et al., 1994), and recent evidence suggests that inhibition of certain PCK isoforms (PKCδ) might help to reduce the progression of the disease in APPswe/PS1dE9 mice (Du et al., 2018).

These results in PKC and AMPA receptor current potentiation suggest a potential involvement in synaptic plasticity, such as the long‐term potentiation phenomenon. Indeed, with extracellular Aβo not only a reduction in LTP has been reported but the opposite phenomenon has also been described at pM and nM concentrations (Puzzo et al., 2008; Wu et al., 1995). Moreover, results from Ling et al. showed that intracellular perfusion of PKMζ (a constitutively active form of PKC) through the patch electrode is sufficient to maintain LTP in hippocampal slices (Ling et al., 2002, 2006). Interestingly, this PKC isoform also increases the potentiation of AMPAergic post‐synaptic currents, a similar effect to the one found in this study with iAβo. Considering this, we believe that based on the characteristics of the physiological response given by neurons exposed to iAβo, it is very likely that long‐term potentiation will be affected because not only is there a greater release of neurotransmitters but also a post‐synaptic potentiation through the increased current mediated by AMPA receptors. The existence of both phenomena is considered favorable for the establishment of synaptic plasticity. Nevertheless, further studies are needed to elucidate this possibility.

PKC has been involved in the functional modulation of the GABA_A_ receptor. For example, it was previously reported that the activation of PKC favors the phosphorylation of beta and gamma‐2 subunits, reducing the activation of the GABA receptor and therefore the amplitude of the current (Kellenberger et al., 1992; Krishek et al., 1994). On the contrary, other studies reported that PKC mediated an increase in cell surface GABA_A_ receptor (Lin et al., 1996; Saliba et al., 2012). In our experiments, iAβo did not alter the post‐synaptic activation of GABA_A_ receptors indicating a differentially regulated effect of iAβo in hippocampal neurons; however, more experiments are needed to clarify the absence of changes in GABA_A_ receptor.

Previous studies showed that not only PKC is able to modulate the function of membrane receptors, but other kinases such as PKA (Protein kinase A) and CAMKII (Ca^2+^/calmodulin protein kinase II) can also alter AMPA and GABA_A_ receptor functions. For example, CAMKII increases AMPA receptor conductance (Lee et al., 2000) and the number of synaptic physical contacts, thus improving the connectivity that mediates excitatory transmission (Pratt et al., 2003). Additionally, it was found that CAMKII increases cell surface expression of GABA_A_ receptors (Houston et al., 2008). On the other hand, PKA can increase the number of AMPA‐R at the synapse (Esteban et al., 2003) and release of neurotransmitters at the pre‐synaptic site (Carroll et al., 1998). Whitcomb and collaborators reported a PKA‐dependent increase in AMPA current amplitude in response to iAβo in hippocampal slices (Whitcomb et al., 2015). However, their results differ from the present study because they required longer times of exposure, and it was calcium‐dependent. These discrepancies may be due to differences in oligomer preparation, time exposure course, or the effective concentration, since in our study the amplitude increased only with higher iAβo concentrations. Interestingly, another study in cultured autaptic hippocampal neurons found that intracellular Aβ decreased neurotransmission by an undetermined mechanism (Ripoli et al., 2014). The present results allow us to conclude that the increase in AMPA receptor function represents a main mechanism by which iAβo depolarizes the post‐synaptic membrane and increases the probability of AP firing.

### 3.3 Implications of pre‐ and post‐synaptic effects at the circuit level in AD pathology: Functional spreading and neural excitability in AD

A quite novel result found during our study was that not only the hippocampal neuron that was dialyzed with iAβo became electrically overactive (Figures 1d, 6d) but that this hyperactivity was transferred to neighboring neurons that displayed increases in NO production in a synchronized fashion with the recorded neuron (Figure 6g). This suggests that there is a phenomenon in which the functional effect of iAβo extends to surrounding neurons in a coordinated way. In other words, by mechanisms only now beginning to be appreciated, iAβo alters the function of hippocampal neurons beyond where it is physically present, thus, iAβo has an impact at the circuit level. Therefore, we have adopted the term "functional spreading" to refer to this phenomenon. Given the critical role of neuronal spiking in the synchronization of the neural network, it is tempting to propose a mechanism that can explain this functional spreading. Because these experiments were performed in the presence of TTX, this synchronicity may depend on other mechanisms, perhaps *via* the activation of the tripartite synapse (Liu et al., 2018). Therefore, further work is needed to explain how this increased level of NO augments activation in surrounding neurons.

From previous studies, it is well known that extracellular release of NO increases the discharge of excitatory neurotransmitters in nearby areas, such as glutamate and D‐serine (Choi & Rothman, 2003; Meldrum, 1994; Paula‐Lima et al., 2013; Rowley et al., 2012), thus increasing neuronal activity, frequency of AP firing, and excitability of the neural network (Balez & Ooi, 2016; Steinert et al., 2010, 2011). Additionally, in an epilepsy model induced by the intrahippocampal injection of kainate, an AMPA receptor agonist, an increase in NOS activity was found suggesting a direct link between NO and AMPA‐mediated epileptiform activity (Yasuda et al., 2008). Although AMPA‐Rs have roles in neuronal physiology [such as plasticity and behavior, for review see (Kessels & Malinow, 2009)], they are also important in epilepsy (Honoré et al., 1988), and AMPA‐R antagonists significantly reduced or nullified epileptiform activity in hippocampal neurons (McBain et al., 1988; Neuman et al., 1988). Thus, our results support the existence of a relationship between NO and increased AMPAergic function in the presence of iAβo contributing to the neuronal excitability that could possibly correlate to the epileptiform activity exhibited by murine AD models and more importantly AD patients (Amatniek et al., 2006; Davis et al., 2014; Hommet et al., 2008; Minkeviciene et al., 2009).

Considering our results, it is possible to hypothesize that only a few neurons with iAβo could initiate and advance the augmentation in neurotransmission and excitability in the whole circuit, initiating an epileptogenic focus and triggering a functional spreading phenomenon for the whole neuronal ensemble with very disrupting consequences to the brain.

Taken together, our study suggests that iAβo exerts two effects (pre‐ and post‐synaptic), both triggered by signal transduction pathways. They could provide a potential mechanism to explain early stages of AD, when iAβo accumulates, increasing neuronal hyperexcitability and becoming an epileptogenic focus at the AD onset. Therefore, the prevention of early effects of iAβo, which are mainly intracellular, can potentially be a new therapeutic target for AD. Early treatment could partially or totally reverse the molecular mechanisms by which the peptide initially triggers the synaptic impairment observed in patients in more advanced stages of this disease.

## 4 EXPERIMENTAL PROCEDURES

### 4.1 Primary cultures of rat hippocampal neurons

Hippocampal neurons were obtained from 18‐day embryos from pregnant Sprague‐Dawley rats and cultured for 10–14 days *in vitro* (DIV) as previously described (Fernández‐Pérez et al., 2018). The use of animals was approved by the Institutional Bioethical Committee of the Universidad de Concepcion in accordance with national guidelines. Where possible, the use of cultured neurons was encouraged to comply with the 3R rules.

### 4.2 Preparation of amyloid beta oligomers

Human Aβ42 fluorescently labeled with FAM (5 (6)–carboxyfluorescein) at the N‐terminal or without fluorescence was bought from Biomatik (USA) and Genemed Synthesis Inc. (USA), respectively. Synthetic oligomeric species of Aβ (later referred as iAβo) were prepared as previously described (Peters et al., 2016). Briefly, Aβ was dissolved in 1,1,1,3,3,3‐hexafluoro‐2‐propanol (HFIP) (10 mg/ml) (Merck Millipore, USA) and incubated in a parafilm sealed tube at 37℃ for 2 h. Then, the solution was incubated at 4℃ for 20 min and centrifuged at 14,000 g for 10 min at 4ºC. Aliquots of 5 µl were placed in 1.5‐ml open lid Eppendorf tubes to allow evaporation. Aliquots were stored at −20℃. To obtain an oligomer‐rich solution, Nanopure water was added to obtain a final concentration of 80 µM and the tubes were incubated at room temperature for 20 min. Subsequently, a Teflon‐coated magnetic stir bar was added to the solution (size: 2x5 mm) and stirred at room temperature (typically 21ºC) at 500 rpm for 24 hrs. Oligomers were freshly prepared on the same day of the experiment and stored at 4ºC until the measurements were performed. Also, to characterize the presence of oligomeric Aβ in the preparations used in all the experiments, we used transmission electron microscopy coupled to immunogold staining that showed the presence of spherical or disk‐shaped structures of Aβ ranging in sizes from 5 to 25 nm approximately (Figure S3c). Furthermore, using this aggregation technique, together with WB, Transmission Electronic Microscopy and AFM analysis, we concluded that most of Aβ are in its soluble oligomeric form, from dimers to small protofibrils (González‐Sanmiguel et al., 2020; Peters et al., 2013, 2016). More importantly, the acute extracellular application of these resulting Aβ species at low concentrations (0.5–1 μM) and dissolved in physiological solution produced an increase in intracellular calcium and synaptic transmission in hippocampal neurons allowing us to conclude that these species are diffusible and interact with cell membranes. Reverse peptide was prepared following the same procedure. Vehicle samples include the solvents used for the preparation of oligomeric species.

### 4.3 Obtaining human‐derived Aβo

Oligomeric assemblies of Aβ were derived from AD brain tissues (referred to as h‐iAβo) by following previously published protocol (Sengupta et al., 2015). Aβo was extracted from the PBS‐soluble fraction of AD brain homogenates using a co‐immunoprecipitation Kit (Thermo Fisher Scientific, USA) following the manufacturer's guidelines. Briefly, amine‐reactive resin was coupled with an anti‐Aβ 6E10 antibody (BioLegend) followed by incubation with the PBS‐soluble fraction of the AD brain homogenate. Bound proteins were eluted in 0.1 M glycine (pH 2.8), and the final pH was adjusted to 7.0 by adding 1 M Tris‐HCl (pH 8). The eluted fraction was subjected to buffer exchange and collected in sterile PBS. This fraction was further separated by size exclusion chromatography using the AKTA Explorer system fitted with a Superdex 200 Increase 10/300 gl Column. Degassed PBS was used as the mobile phase with a flow rate of 0.5 ml/min to collect the Aβo fraction. The total protein concentration was measured with a bicinchoninic acid protein assay (Pierce™ Micro BCA Kit, Thermo Fisher Scientific, USA). Human brain‐derived Aβo was characterized by Western blot analysis and atomic force microscopy.

### 4.4 Electrophysiology

Whole‐cell recordings were used to apply Aβ peptide intracellularly through the internal solution contained in the recording electrode and simultaneously record post‐synaptic currents at constant voltage (voltage‐clamp) or APs (current‐clamp) *in vitro*, *ex vivo*, and/or *in vivo*. We intended to use the largest pipette tips possible (pipette resistance 4–5 MOhm, access resistance <15 MOhm) to achieve fast iAβo dialysis (Pusch & Neher, 1988), but this usually hampered recordings from small central neurons (<15 μm). The access resistance was compensated by >80%, and cells that showed large variations (>15%) were discarded. *In vitro* cultured hippocampal neurons, composed of a mix of interneurons and projecting pyramidal neurons, were used to characterize mechanisms of action of intracellular Aβ that required the recording of a large number of cells, while *ex vivo* or *in vivo* experimental methodologies were used to confirm critical actions of Aβ using a more complex and mature neuronal network.

#### 4.4.1 Voltage‐clamp experiments *in vitro* and *ex vivo*

For *in vitro* experiments, the dish culture medium was replaced with a normal external solution (NES) containing (in mM): 150 NaCl, 5.4 KCl, 2.0 CaCl_2_, 1.0 MgCl_2_, 10 glucose, and 10 HEPES in deionized water with a resistance of 18.2 MΩ · cm at 25 ºC (referred to as DI water), pH 7.4 adjusted with NaOH, 310 mOsm/L. Cells were stabilized at room temperature for 20 min before beginning the experiments. Unless otherwise noted, the internal solution used to record voltage‐clamp experiments, including synaptic currents or ligand‐evoked currents, contained (in mM): 120 KCl, 2.0 MgCl_2_, 2 Na_2_ATP, 10 BAPTA, 0.5 NaGTP, and 10 HEPES (pH 7.4 adjusted with KOH, 290 mOsm/L). To study miniature post‐synaptic currents (mPSC), 500 nM tetrodotoxin (TTX, a sodium channel blocker) (Hello Bio) pre‐dissolved in DI water was applied in the NES of the well containing the cells. To record excitatory and inhibitory spontaneous PSC (sEPSC and sIPSC), the voltage‐dependent intracellular sodium channel inhibitor QX‐314 was used (Tocris, USA). QX‐314 was dissolved in DI water as a 100 mM stock and applied at 5 mM in the following internal solution (in mM): 114 K‐Gluconate, 4 KCl, 4 MgCl_2_, 10 BAPTA, and 10 HEPES (pH 7.4 adjusted with KOH, 290 mOsm/L). This was later referred to as “*low Cl* ^−^ *internal solution*.” The recordings for sEPSC and sIPSC were done at a voltage holding of −60 mV and +10 mV, respectively. For the AMPAergic evoked (eEPSC) and GABAergic (eIPSC) currents, 500 nM of TTX was used in the NES solution. 100 µM of extracellular α‐amino‐3‐hydroxy‐5‐methyl‐4‐isoxazolepropionic acid (AMPA) (Hello Bio, UK) and γ‐aminobutyric acid (GABA) (Sigma, Germany) pre‐dissolved in DI water and then in NES were applied via lateral motion of a gravity‐driven multi‐pipette array (2–3 ml/min) during 2 or 3 s to evoke the current and then washed thoroughly using the perfusion system. Additionally, the external solution was continuously replaced to avoid unnecessary accumulations of these neuroactive molecules in the well during the recording period. To isolate the AMPAergic miniature currents (mEPSC) *in vivo* and *ex vivo*, synaptic transmission inhibitors were applied using the same perfusion system (in µM): 20 2‐amino‐5‐phosphonopentanoate (DAPV), 1 strychnine, and 10 bicuculline. The same approach was used to isolate GABAergic miniature currents (mIPSC) *in vitro* and *ex vivo* by perfusing (in µM): 20 DAPV, 1 strychnine, and 20 6‐Cyano‐7‐nitroquinoxaline‐2,3‐dione (CNQX). All synaptic transmission inhibitors were purchased from Tocris, USA. For recordings of synaptic currents in CA1 hippocampal brain slices (*ex vivo*), the rats were sedated with isoflurane and decapitated. The brain was removed, and coronal hippocampal cuts of 300–400 µm thick were made in a VT1200S vibratome (Leica) in a cold solution containing (in mM): 194 Sucrose, 30 NaCl, 4.5 KCl, 1 MgCl_2_, 26 NaHCO_3_, 1.2 NaH_2_PO_4_, and 10 Glucose. Once the slices were obtained, they were allowed to stand in a chamber at room temperature (22℃) for 1 h in artificial cerebrospinal fluid (aCSF) bubbling with 95% O_2_ and 5% CO_2_. The aCSF solution contained (in mM): 120 NaCl, 4.5 KCl, 1 MgSO_4_, 2.5 CaCl_2_, 1 NaH_2_PO_4_, 25 NaHCO_3_, and 20 glucose. The slices were then transferred to the recording chamber with aCSF solution saturated with 95% O_2_ and 5% CO_2_ and continuously perfused with oxygenated aCSF at a rate of ∼2 ml/min at room temperature (RT). Whole‐cell voltage and current‐clamp recordings were made using an Axopatch 200B amplifier (Axon Instruments, USA) and Digidata 1322A (Molecular Devices). All recordings were filtered at 2.2 kHz and digitized at 10 kHz. Data were acquired using Clampex 10 software (Molecular Devices). Series resistance was continuously monitored, and only cells with a stable access resistance (less than 15% variation) were included for data analysis. All currents (synaptic and evoked) were recorded in voltage‐clamp mode by adjusting the membrane potential to −60 mV (unless otherwise noted). Voltage‐clamp *in vitro* experiments were performed using an Axopatch 200B amplifier (Molecular Devices, USA) and an inverted microscope (Nikon Eclipse TE200‐U). The acquisition was made using a computer connected to the recording system using a Digidata 1440A acquisition card (Molecular Devices, USA) and the pClamp10 software (Molecular Devices). Electrodes with a resistance of 4–5 MΩ were pulled from borosilicate capillaries (WPI) in a horizontal puller (P1000, Sutter Instruments).

#### 4.4.2 Current‐clamp experiments *in vitro*

To study the membrane potential (*V*_m_), recordings were made in current‐clamp mode as previously described (Förstera et al., 2017) using the previously mentioned *low Cl* ^−^ *internal solution*. To evoke APs, a family of current pulses applied for 300 ms was used (from −300 pA to +275 pA, increasing by 25 pA steps). Some experiments involved the use of QX‐314. Before starting the recording of evoked APs, a small holding current (−2 to ‐ 50 pA) was applied to stabilize the resting membrane potential (RMP) to −70 mV. Current‐clamp *in vitro* experiments were performed using an Axopatch 200B amplifier (Molecular Devices) and an inverted microscope (Nikon Eclipse TE200‐U).

#### 4.4.3 Current‐clamp recordings *in vivo*

All experiments on live animals were approved by the Institut National de la Santé et de la Recherche Médicale (INSERM) Animal Care and Use Committee, in accordance with the guidelines of the European Community Council directives (2010/63/EU). Data were obtained from male Wistar rats between the ages of postnatal day 25 (P25) to P35 (weight range, 90–110 g). Recordings were made as previously described (Morgan et al., 2019). On the day of the recordings, the animals were anesthetized (induction: 3% isoflurane; maintenance: Xylazine/Ketamine 10/100 mg/Kg, supplemented with ketamine 20 mg/Kg). The level of anesthesia was assessed by pinching the foot and by measuring body temperature and respiratory rate. Body temperature was maintained at 37℃ with a thermal blanket (FHC). The animals were fixed in a stereotactic apparatus (SR‐6, Narishige). A local analgesic (lidocaine) was applied as a gel on the stereotaxic system bars to reduce pain during fixation of the head with the stereotaxic system bars, and it was also injected as a liquid under the skin before the first incision. An ophthalmic gel was applied to the eyes to prevent them from drying out during surgery, and the eyes were covered with a piece of cardboard to protect them from light during surgery. The skull was exposed, and two small craniotomies (2 mm in diameter) were perforated on both hippocampus (−3.5 mm posterior to bregma; 2.5 mm lateral to bregma) to record in the CA1 area (3 mm deep from the surface of the brain). The *V*_m_ of CA1 neurons was recorded in the current‐clamp mode, using standard techniques for "blind patch" blind cell clamp *in vivo* (Lee et al., 2009). Before starting the recording of evoked APs, a small holding current was applied to stabilize the resting membrane potential (RMP) to −70 mV and evoked APs were recorded as described in Section 2.4.2. The borosilicate electrodes that were used had a resistance of 5–7 MΩ. The internal solution contained (in mM): 135 K‐Gluconate, 5.4 KCl, 10 HEPES, 2 Mg‐ATP, 0.4 GTP, 0.2 EGTA, and 0.2% of biocytin (pH 7.2, adjusted with KOH). The *V*_m_ was amplified by an NPI ELC‐03XS amplifier (NPI Electronics, Germany) and digitized with a LIH (HEKA Electronik), using Patch Master software (HEKA Electronik, Germany). Finally, output signals were digitized with a 1440A Digidata (Molecular Devices, USA) and recorded with Axoscope software (Molecular Devices). 50 Hz noise was removed using a HumBug noise eliminator (Quest Scientific). For further analysis, only cells with *V*_m_ at rest under −55 mV were considered.

### 4.5 Histology

At the end of the recording period and to confirm the location and the morphology of *in vivo* recorded neurons, we performed immunohistochemical analysis of hippocampus sections. Animals were injected with a ketamine overdose and transcardially perfused with 1x PBS solution followed by 4% paraformaldehyde fixation. The brains were then left in 4% PFA overnight at 4˚C, before washing and storing at 4˚C in PBS. The next day, 50 µm thick coronal slices were *post hoc* processed with the streptavidin method associated with the Cy3 fluorophore (Jackson ImmunoResearch) to visualize neurons containing biocytin. For this, brain slices were incubated with PBS containing 0.3% Triton X‐100 (Sigma), 2% normal goat serum (Thermo Fisher Scientific, USA), and 1:1000 Cy3^TM^ streptavidin (Jackson ImmunoResearch) for 48 – 72 hrs at 4˚C (continuously agitated, and protected from light). After confirming the location of the recorded cell in the hippocampus, slices were blocked with PBST (PBS and 0.3% Triton X‐100) plus 7% normal goat serum for 2 hrs at 4˚C, continuously agitated and protected from light. Immunostaining was performed using a rabbit anti‐calbindin–D‐28k antibody diluted 1:1000 (Swant) in a solution containing: PBS, 0.3% Triton X‐100, 2% normal goat serum, and 1:1000 Cy3^TM^ streptavidin for 24 hrs at 4˚C (continuously agitated and protected from light). Slices were washed with PBST (3 times per 10 min at RT, continuously agitated and protected from light) and then incubated with a secondary Alexa Fluor® 488 Donkey Anti‐Rabbit antibody diluted 1:1000 (Jackson ImmunoResearch) using the same protocol and solution of the primary antibody. After washing with PBST (3 times per 10 min at RT, continuously agitated and protected from light) and PBS (2 times per 10 min at RT, continuously agitated and protected from light), samples were mounted with VECTASHIELD mounting medium (Vectorlabs). Eight bit images were obtained using a confocal upright Leica TCS SP5 X microscope (Leica) with a 40x oil immersion objective (1.3 NA) and under the following conditions: For excitation, we used 2 laser lines (488 nm, 555 nm), and emission was collected in the 490–540 nm and 569–610 nm ranges, respectively (example in Figure 7).

### 4.6 Simultaneous recordings of electrophysiology and fluorescence

Simultaneous studies were performed using the same methodology described for the recording of synaptic currents in voltage‐clamp mode *in vitro* (Section 2.4.1) together with NO fluorescence using a previously described methodology to detect NO using 1,2‐aminoanthraquinone (DAQ) (Sigma, Germany) (Chen, 2001; Galindo et al., 2008; Schuchmann et al., 2002; von Bohlen und Halbach et al., 2002). DAQ was pre‐dissolved in DMSO at 2.5 mg/ml, and hippocampal neurons were incubated with DAQ at a final concentration of 2.5 µg/ml in NES for 20 min at 37℃ (<0.1% DMSO). Neurons were washed three times with NES and mounted in a well on an inverted microscope (TE200‐U, Nikon, USA) equipped with a 16‐bit IonXEM CCD camera (Andor), a 20X/0.4 NA objective (Nikon, Japan) and a voltage‐clamp configuration for *in vitro* studies. The fluorescent signal for the DAQ probe was obtained by exciting with a bandpass filter (528–553 nm) and collecting the fluorescence with a bandwidth emission filter (590–650 nm) (Nikon, USA). Image acquisition was performed with a computer‐controlled Lambda 10‐B shutter (Sutter Instruments) using Imaging Workbench 5.0 software (INDEC BioSystems) and exciting for a period of 900 ms at intervals of 1 s during a continuous period of 20 min. Some experiments involved the use of other molecules: Nω‐Nitro‐D‐arginine methyl ester hydrochloride [L‐NAME, an NO synthase (NOS) inhibitor] (Sigma), 1400W dihydrochloride [inducible NOS (iNOS) inhibitor] (Tocris), S‐nitroso‐*N*‐acetyl‐dl‐penicillamine (SNAP, an NO donor) (Sigma), and 2‐(4‐carboxyphenyl)‐4,4,5,5‐tetramethyl‐imidazoline‐l‐oxyl‐3‐oxide as potassium salt (CPTIO, a highly specific NO scavenger) (Cayman Chemical) (Akaike et al., 1993). Fresh stocks of all of these reagents dissolved in DI water were prepared on the same day that the experiment was performed. For experiments that involved NOS inhibition, cells were pre‐incubated with 200 µM L‐NAME for 20 min in NES before fluorescence and patch‐clamp recordings. Since the effects of this drug are very reversible, we maintained the same L‐NAME concentration in the wells containing the cells. To inhibit iNOS, cells were pre‐incubated with 1400W 200 µM for 24 h before proceeding with recordings. Finally, 200 µM CPTIO was pre‐incubated for 15 min for the scavenging of NO, and bath‐application of 300 µM SNAP was used as an NO donor at the moment of the recordings.

### 4.7 Western Blot

Human brain‐derived Aβo was characterized by Western blot analysis. Two different concentrations of Aβo (1 and 0.5 µg of protein) were loaded onto precast NuPAGE 4–12% Bis‐Tris gel (Invitrogen) for SDS‐PAGE analysis. The gel was subsequently transferred onto nitrocellulose membranes and blocked with 10% nonfat dry milk at 4℃ overnight. The membrane was then probed with primary antibodies, A11 (1:1000) and anti‐Aβ_1−17_ 6E10 (1:6000, BioLegend, USA) diluted in 5% nonfat dry milk for 1 h at RT. HRP‐conjugated anti‐rabbit IgG and anti‐mouse IgG (1:6000, Cytiva) were used to detect A11 and 6E10 immunoreactivity, respectively. ECL plus (Cytiva) was used to visualize the bands.

### 4.8 Atomic force microscopy

Human brain‐derived Aβo was also analyzed by AFM using a non‐contact tapping method with a Multimode 8 AFM machine (Bruker). Briefly, 3–4 µl of Aβo was applied onto a fresh‐cleaved mica surface and allowed to adsorb at RT overnight. Mica was then washed with 200 µl of deionized water, air‐dried, and imaged.

### 4.9 Immunogold and negative contrast transmission electron microscopy

Five microliters of Aβo, at a concentration of 50 μM, were applied to carbon‐coated Formvar grids (Agar Scientific). Nonspecific immunoreactivity was blocked with 3% bovine serum albumin (BSA) for 30 min at room temperature and incubated with the primary antibody anti‐Aβ 6E10 (1:50; Novus Biologicals) for 1 h. A secondary 5‐nm gold‐conjugated anti‐mouse IgG antibody (Merck) was used at a 1:20 dilution for 30 min. Samples were fixed with a 2% glutaraldehyde solution for 5 min. Aβo was stained with 5 μl of 0.2% (wt/vol) phosphotungstic acid, and the grid was air‐dried. Samples were examined using a JEOL 1200 EX II electronic microscope.

### 4.10 Data analysis

Synaptic current parameters (frequency and amplitude) were analyzed using Mini analysis software (Synaptosoft, Inc.) that identifies the currents based on several criteria such as the amplitude, the area under the curve, and the decay time of each event. As a routine check, we visually inspected all events detected by the software and rejected any that did not exhibit the general expected form for synaptic events. 10 pA was used as a threshold to detect synaptic currents. For spontaneous synaptic recordings, the area under the current trace was integrated (pA · ms) and expressed as charge transferred (nC) during the whole recording (2 min) using Clampfit 10.5 (Molecular Devices, USA). We quantified five continuous blocks of 2 min (total =10 min). For the I/E balance experiments (Figure 3), the analysis was similar, but the baseline current was not included in the analysis. We determined that about 100 events in total were quantified per neuron for the miniature currents. AP parameters were calculated in the first spike of the response as follows: Threshold was numerically estimated from first derivative in a V’ versus V phase space projection. From this value, amplitude was calculated to the maximum value reach by the AP waveform. Finally, we obtained the half width of the AP peak expressed as duration. Input resistance was obtained from the slopes in V/I curves in hyperpolarizing current steps. Rheobase was extrapolated from spikes vs. injected current curves using Origin 2019b (Origin Lab). Spontaneous spike firing frequency was obtained using pClamp10 software (Molecular Devices). All data obtained from all parameters were plotted using Origin 2019b (Origin Lab). Data are shown as mean ± SEM for normally distributed populations and as median and interquartile ranges (IQR) for non‐normally distributed populations. Statistical analyses were performed using the two‐tailed unpaired Student's *t* tests (α = 0.05) or the two‐tailed Mann–Whitney *U* test (α = 0.05) as appropriate, after testing for normality with Shapiro–Wilk test (for *n* < 50) or Kolmogorov–Smirnov (for n>50) and for homogeneity of variances with Levene's test. Data with more than two groups or factors were analyzed by two‐way ANOVA test (*α* = 0.05). One‐way ANOVA test was used to compare several populations of neurons, followed by Tukey or Welch's ANOVA with Games–Howell *post hoc* test to correct for variance heterogeneity using R software (R Development Core Team 3.0.1., 2013) (www.r‐project.com. A probability level (*p*) < 0.05 was considered statistically significant (* *p* < 0.05, ** *p* < 0.01, *** *p* < 0.001). Unless otherwise noted, all “n” values represent individually recorded neurons and are given in the legend of each figure for each condition.

## CONFLICT OF INTEREST

The authors declare that they have no competing interests.

## AUTHOR CONTRIBUTIONS

EJFP, RK, JE,and LGA designed experiments and discussed the results. EJFP and LGA contributed to all stages of manuscript preparation and editing. Material preparation, data collection, and analysis were performed by EJFP, BM, DAB, CP, NORL, MPE, JPM, CF, RB, US. All authors read and approved the final manuscript.

## ETHICS APPROVAL

All experimental procedures were in accordance with Institutional Animal Care and Use Committee guidelines for animal research at the University of Concepción.

## Supporting information

Supplementary Material

## Data Availability

The datasets used and/or analyzed during the current study are available from the corresponding author on reasonable request.
