# Corrigendum: Synaptic dysregulation and hyperexcitability induced by intracellular amyloid beta oligomers

**DOI:** 10.1111/acel.13495

**Published:** 2021-10-21

Fernandez‐Perez, E. J., Muñoz, B., Bascuñan, D. A., Peters, C., Riffo‐Lepe, N. O., Espinoza, M. P., Morgan, P. J., Filippi, C., Bourboulou, R., Sengupta, U., Kayed, R., Epsztein, J., & Aguayo, L. G. (2021). *Aging Cell*, **20**, e13455. https://doi.org/10.1111/acel.13455

In the paper by Fernandez‐Perez et al. (2021), an additional affiliation ‘Turing Center for Living Systems (CENTURI), Marseille, France’ was included to the co‐author Jérôme Epsztein.

