# Supplementary material for: Synaptic dysregulation and hyperexcitability induced by intracellular amyloid beta oligomers
Source: Aging Cell. 2021 Aug 19;20(9):e13455. doi: 10.1111/acel.13455 (PMC8441418; doi:10.1111/acel.13455)
Supplement: Supplementary file 1 — Supplementary Material [file ACEL-20-e13455-s001.pdf]

Additional file 1

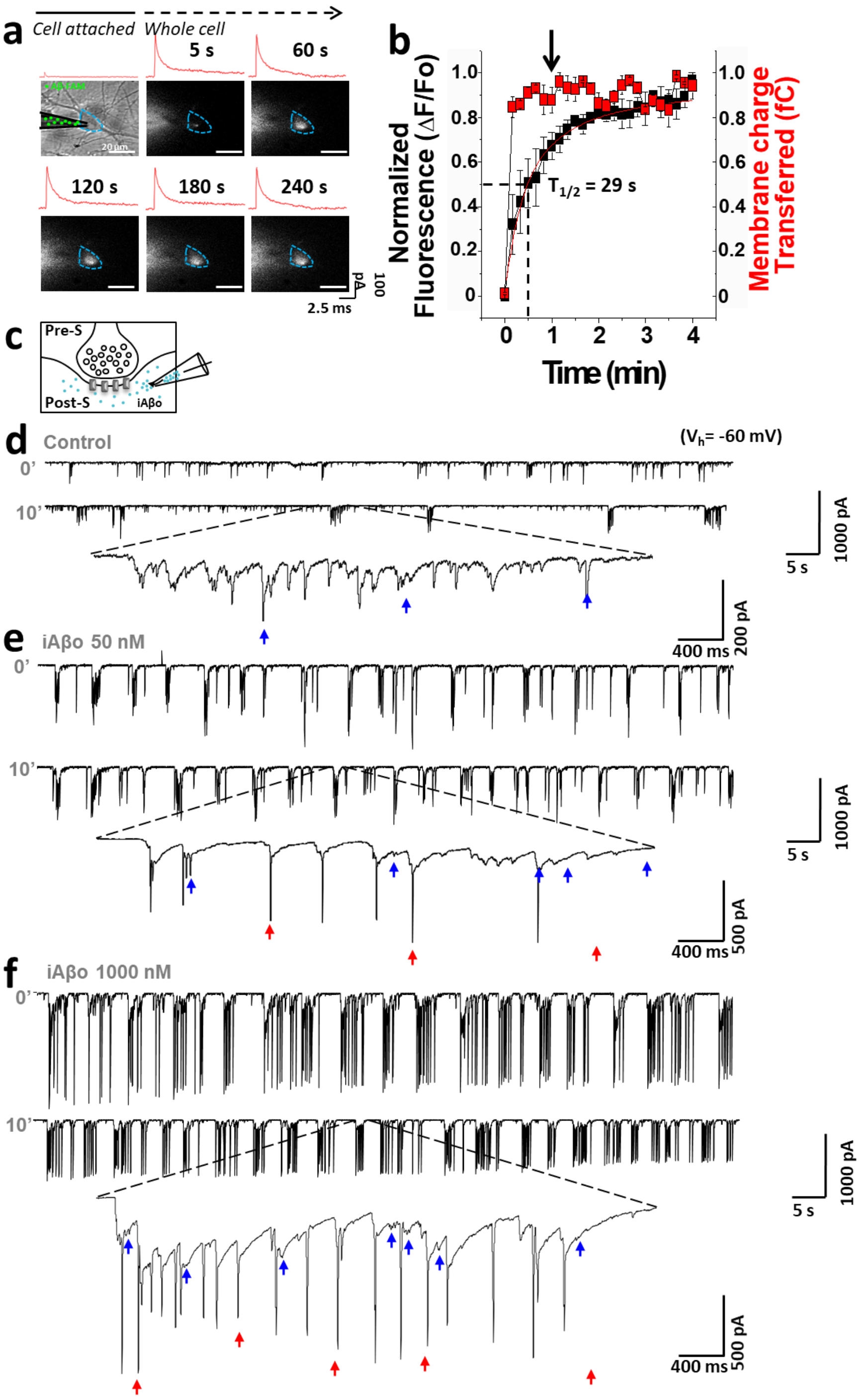

Figure 1. The whole cell technique allows rapid entry of fluorescent Aβo into the intraneuronal compartment. a, Simultaneous registration of patch clamp and fluorescence showing the entry of fluorescently labeled Aβo (in green) from inside the recording electrode to the intracellular medium. The region of interest (ROI) in (in light blue) delimits the contour of the recorded neuron. Fluorescence quantification was carried out within this region and it was observed that increases inside the cell throughout the experiment (0-4 min). Along with this, the traces of the capacitive currents recorded at different intervals (in red) are observed. **b,** Quantification of the fluorescence in the ROI previously described, together with the membrane charge transferred. The latter reflects that the solution contained in the patch pipette instantly reaches the intracellular compartment, while the fluorescence accounts for a gradual entry of the peptide, reaching 50% of the total fluorescence value at 29 s ($T_{1/2}$). The black arrow indicates the time at which synaptic currents began to be recorded. **c,** Schematic representation of the synaptic recording, showing the pre-synaptic (Pre-S) and post-synaptic (Post-S) compartment, and the application of iAβo in the latter using the patch electrode (orange squares represent post-synaptic receptors). **d, e, f,** Total synaptic recordings obtained at the beginning (time = 0') and end of the experiment (time = 10'), demonstrating a rapid and marked increase in the frequency and amplitude of synaptic currents as the concentration of intracellular iAβo oligomers (iAβo) increases from 50 nM (**e**) to 1000 nM (**f**) (V_h = -60 mV). Bursts of synaptic currents (arrows in blue) and spikes in current-recording mode are indicated by red arrows, which increase as the concentration of iAβo in the recording electrode augmented. Line charts represent the average ± SEM. n = 18 cells per condition.

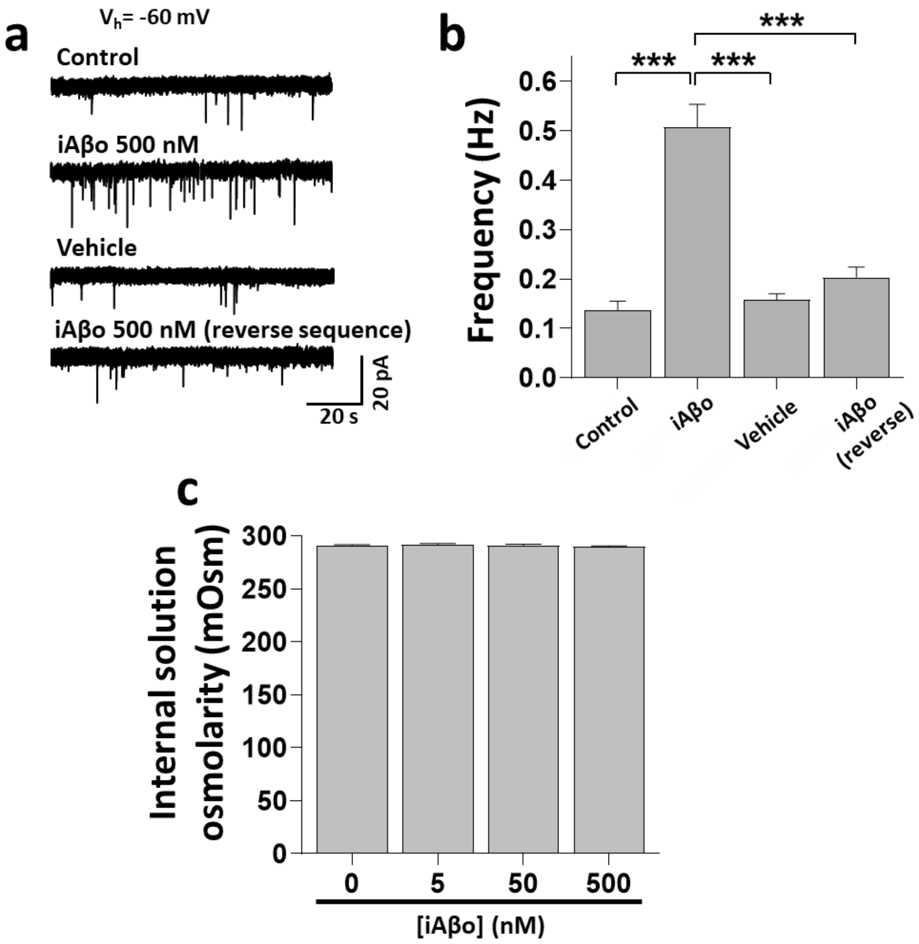

Figure 2. Reverse iAβo does not have an effect on frequency of miniature post-synaptic currents *in vitro*. a, b, Representative mPSCs traces (**a**) and frequency quantification (**b**) of iAβo, reverse sequence and vehicle controls (One-Way Welch's ANOVA with Games-Howell post-hoc test for: $F(3,16.79)=18.78$, $p=1.30E-5$. p-values for post-hoc test: control vs. iAβo 500 nM: 1.07E-4, iAβo vs. vehicle: 2.49E-4 and iAβo vs. iAβo reverse: 4.97E-4). **c,** Internal solution osmolarity measurements show no change when adding different concentrations of iAβo. Bar charts represent the average ± SEM for control (n=9), iAβo (n=9), vehicle (n=9) and iAβo reverse (n=9) cells. *** denotes p <0.001.

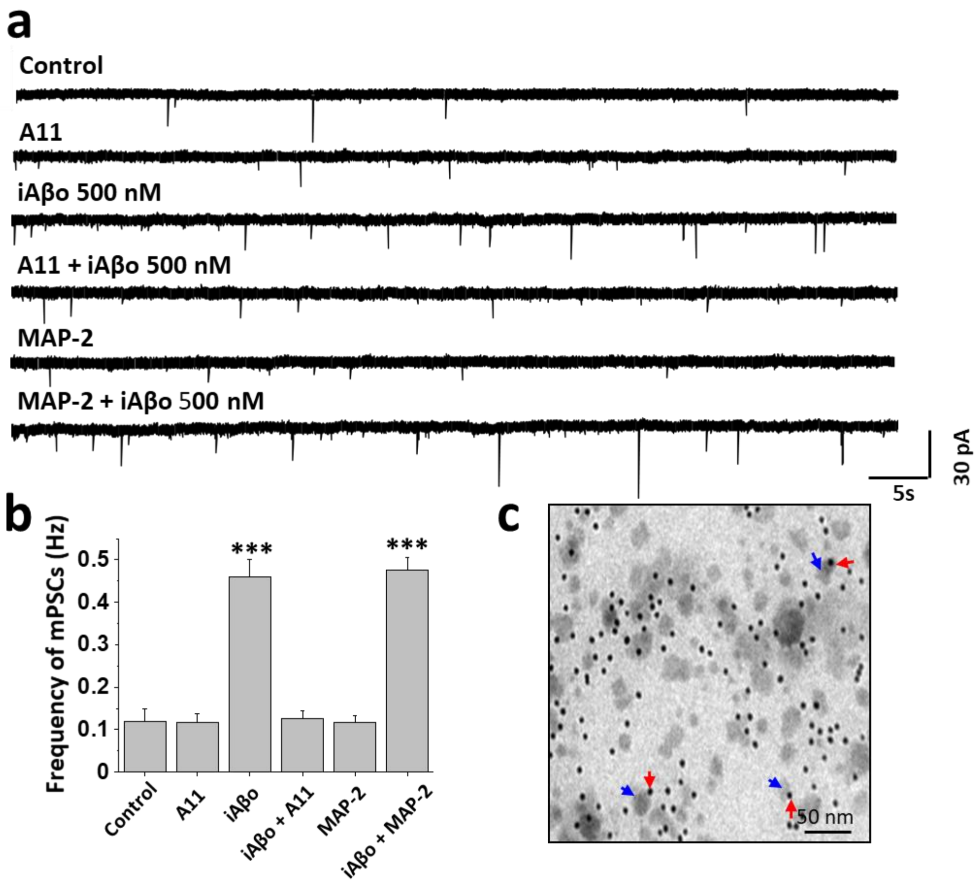

Figure 3. Pre-incubation with A11 antibody attenuates the intracellular synaptic effect of iAβo on the frequency of miniature post-synaptic currents *in vitro*. a, Representative traces showing the increase in the frequency of mPSCS after application of iAβo 500nM alone or pre-incubated for 10 min with antibody A11 or MAP-2. Intracellular dialysis of the antibodies did not have an effect per se on the frequency of mPSCs (V_h = -60 mV). **b,** Quantification of the frequency of mPSCs under the conditions described in **a**, showing that iAβo increases the frequency of miniature synaptic currents, but this effect is diminished to control levels in a similar way with A-11 pre-incubation. No differences in the effect of iAβo are observed when pre-incubating with an antibody for MAP-2 (one-way ANOVA with Tukey post-hoc: $F(5,44)=27.436$, $p=1.7E-12$). **c,** Electronic micrographs demonstrating the presence of amyloid oligomeric aggregates (arrows in blue) in the preparations used. Along with that, the presence of gold nanoparticles coupled to the secondary antibody used for Aβ immunodetection is also observed (arrows in red). Bar charts

represent the average ± SEM for control (n=8), A11 (n=8), iAβo (n=9), iAβo+A11 (n=9), MAP-2 (n=9) and iAβo+MAP-2 (n=7) cells. *** denotes $p < 0.001$.

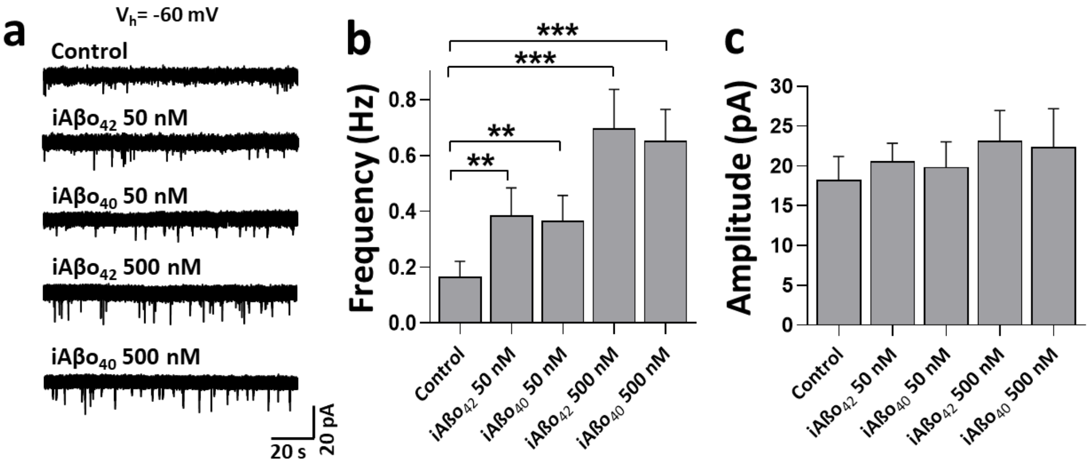

Figure 4. Effects of synthetic intracellular Aβ42 and Aβ40 oligomers on the frequency and amplitude of miniature post-synaptic currents in vitro. a, mPSC recordings of primary hippocampal neurons obtained in presence of TTX 500 nM for control, iAβo42 (50 and 500 nM) and iAβo40 (50 and 500 nM) (n=8 cells per condition). **b, c,** mPSC frequency (**b**) and amplitude (**c**) quantification for each of the conditions described in (**a**). Bar charts represent the average ± SEM. One-Way Welch's ANOVA with Games-Howell post-hoc test for (**b**): $F(4,16.95)=44.08$, $p= 9.6E-9$. p-values for post-hoc test: control vs. iAβo42 50 nM: 1.39E-3, control vs. iAβo40 50 nM : 1.46E-3, control vs. iAβo42 500 nM : 2.21E-5, control vs. iAβo40 500 nM: 4.16E-6. One-Way Welch's ANOVA with Games-Howell post-hoc test for (**c**): $F(4,17.24)=2.269$, $p=0.103$. p-values for post-hoc test: control vs. iAβo42 50 nM: 0.4276, control vs. iAβo40 50 nM : 0.8381, control vs. iAβo42 500 nM : 0.0786, control vs. iAβo40 500 nM: 0.2929. ** denotes $p <0.01$ and *** $p <0.001$.

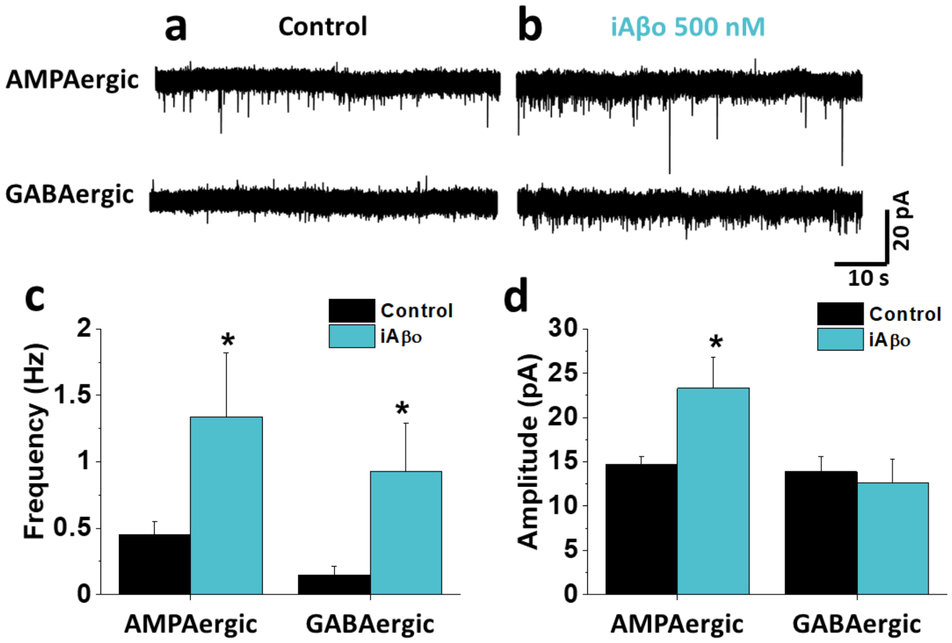

Figure 5. iAβo increases the frequency of AMPAergic and GABAergic synaptic currents in CA1 hippocampal neurons *ex vivo*. a, b, Representative AMPA and GABA mPSCs obtained in acute hippocampal slices for control condition and with iAβo 500 nM, respectively (V_h = -60 mV). **c,** Quantification of the frequency for AMPAergic (unpaired Student's t-test with Welch's correction: t(6.54)=-2.306, p =3.39E-2) and GABAergic (unpaired Student's t-test with Welch's correction: t(6.40)=-2590, p=1.97E-2) mPSCs. **d,** Amplitude quantification for AMPAergic (unpaired Student's t-test with Welch's correction: t(10.32)=-2.540, p=1.95E-2) and GABAergic (unpaired Student's t-test with Welch's correction: t(10.94)=0.394, p=7.01E-1) miniature currents. Bar charts represent the average ± SEM for control (n=12) and iAβo (n=7) cells. * denotes p <0.05.

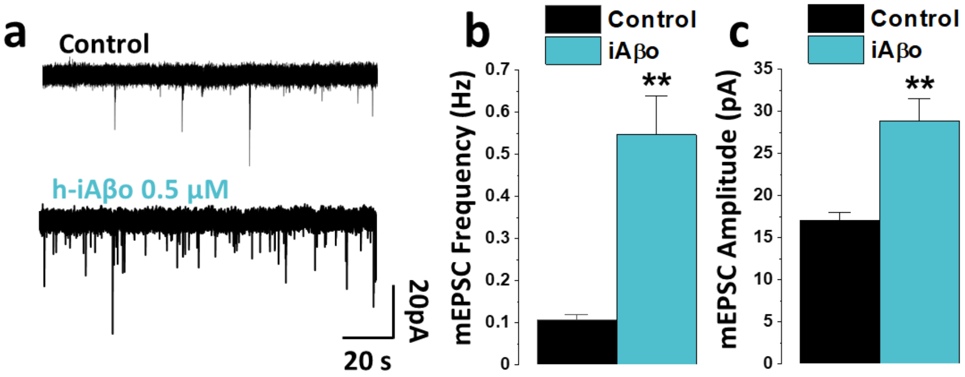

Figure 6. h-iAβo increased AMPA-R mediated mEPSCs *in vitro*. a, Representative traces of AMPA mEPSCs in control condition and with intracellular application of h-iAβo 0.5 µM (V_h = -60 mV). **b, c,** Quantification of the frequency (**b**) (n=7) (t(7.29)=-4.686, p=2.01E-3) and amplitude (**c**) (n=8) (t(8.73)=-4.323, p=2.03E-3) of the AMPAergic miniature currents, demonstrating a significant increase in presence of h-iAβo. Scatter plots represent the average ± SEM. Unpaired Student's t-test with Welch's correction for (**b**) and (**c**). ** denotes $p < 0.01$.

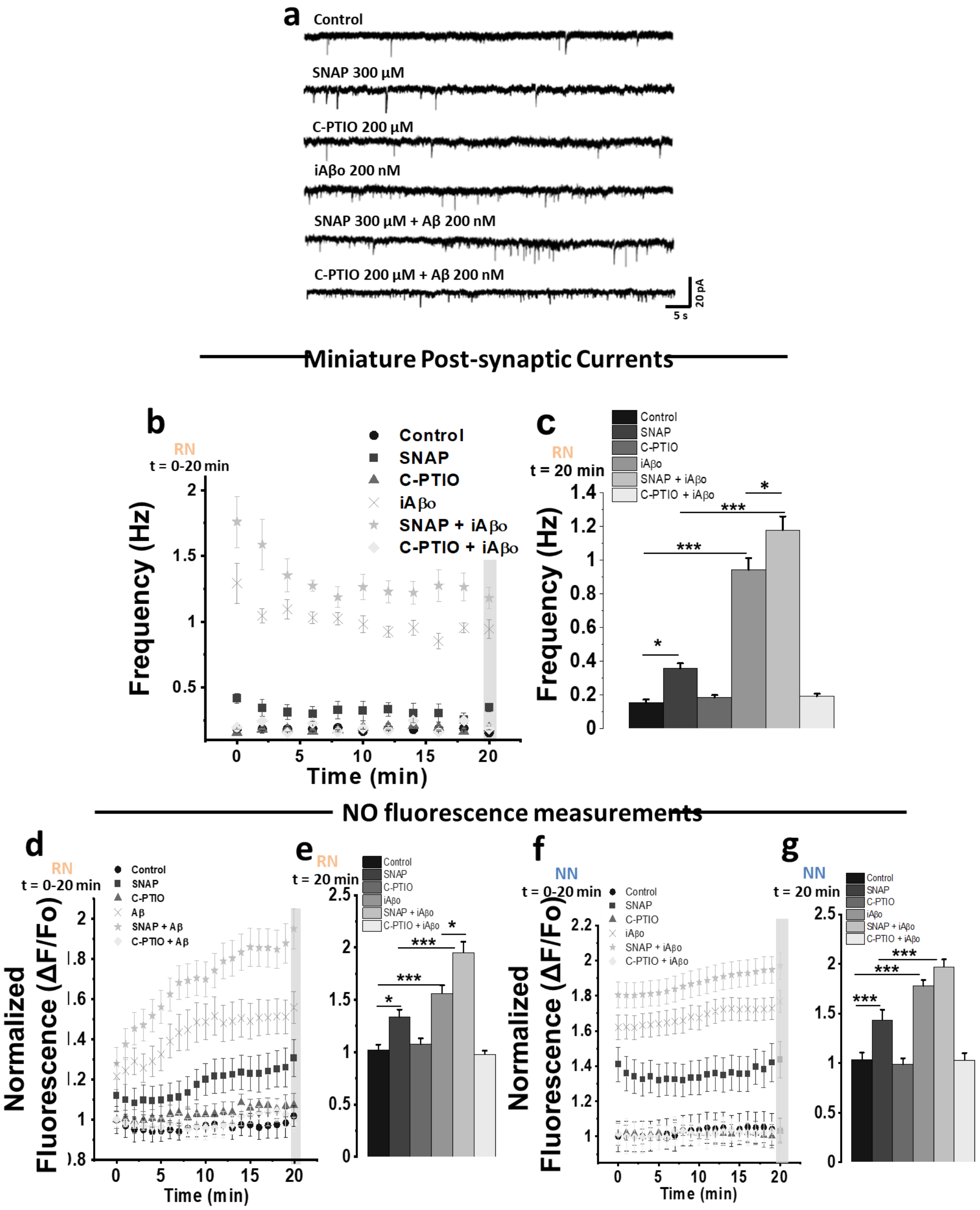

Figure 7. Nitric oxide is involved in the pre-synaptic retrograde signaling of iAβo on the frequency of miniature synaptic currents. a, Representative mPSCs traces obtained in absence and presence of Aβ or 200 nM using a NO donor molecule (300 µM SNAP, in gray) or NO scavenger (C-PTIO 200 µM) (V_h = -60 mV). It is observed that SNAP per se has an effect on the frequency of synaptic currents, but when co-applying iAβo+SNAP this effect increases

considerably, even exceeding the effect that iAβo has on its own. On the contrary, the application of C-PTIO did not affect the frequency of mPSCs, but co-applied with iAβo decreased the frequency to control levels. **b,** Time-course of mPSCs frequency from 0 to 20' in all the experimental conditions described in **a**. **c,** Bar graph represents the data recorded at time 20' obtained from graph **b**. **d - g,** Relative levels of NO (expressed as fluorescence) obtained throughout the course of the experiment and at 20' for RN (**d** and **e**) and NN (**f** and **g**). Bar and line charts represent the average ± SEM. Control (n=6), SNAP (n=6), C-PTIO (n=6), iAβo (n=6), iAβo+SNAP (n=6), iAβo+C-PTIO (n=6) for RN and Control (n=57), SNAP (n=51), C-PTIO (n=49), iAβo (n=56), iAβo+SNAP (n=52), iAβo+C-PTIO (n=53) for NN. One-way ANOVA with Games-Howell comparison for (**c**): $F(5,30)=89.902$, $p=3.49E-23$. p-values for post hoc test: Control vs. SNAP: 4.09E-02, Control vs. iAβo: 3.17E-13, SNAP vs. SNAP + iAβo: 7.56E-14 and iAβo vs. iAβo + SNAP: 4.29E-2. One-Way Welch's ANOVA with Games-Howell post-hoc test for (**e**): $F(5,30)=34.685$, $p=1.34E-11$. p-values for post hoc test: Control vs. SNAP: 4.18E-02, Control vs. iAβo: 1.38E-4, SNAP vs. SNAP + iAβo: 5.84E-7 and iAβo vs. iAβo + SNAP: 2.27E-4. One-Way Welch's ANOVA with Games-Howell post-hoc test for (**g**): $F(5,312)=36.376$, $p=2.57E-29$. p-values for post hoc test: Control vs. SNAP: 4.26E-6, Control vs. iAβo: 9.10E-13, SNAP vs. SNAP + iAβo: 2.98E-4. * denotes $p < 0.05$, *** $p < 0.001$.

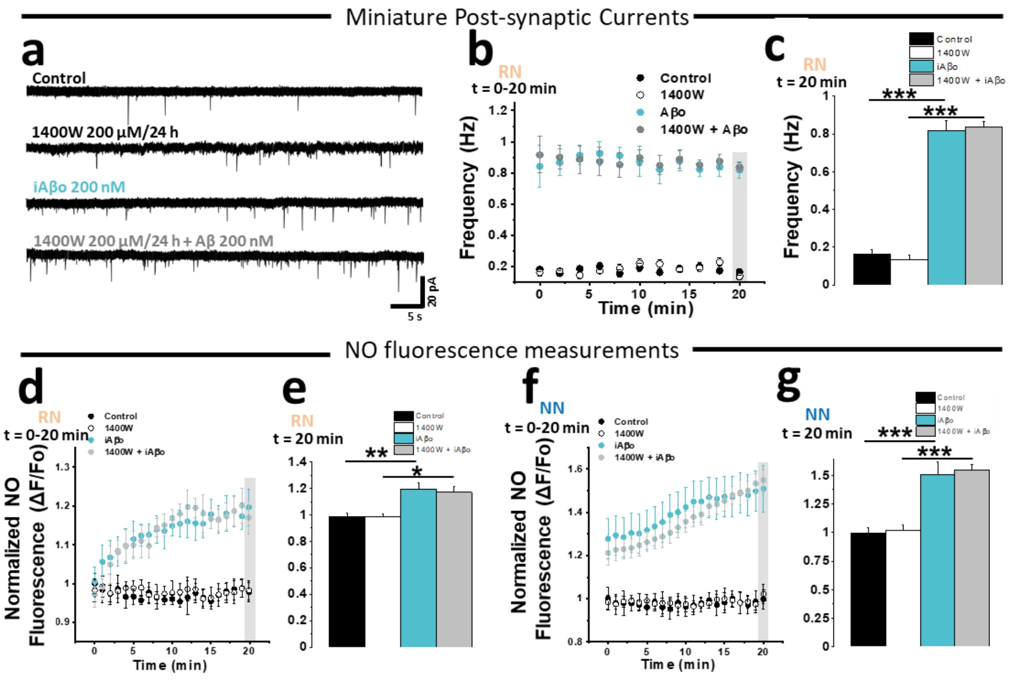

Figure 8. iNOS inhibitor does not affect iAβo actions on synaptic current frequency. a - c, Representative recordings and quantification of the frequency of miniature post-synaptic currents in absence and presence of iAβo or 200 nM, together with the co-application of an iNOS inhibitor (1400W) 200 µM for 24 hours (V_h = -60 mV). It is observed that 1400W *per se* does not have an effect on the frequency of synaptic currents. On the other hand, by pre-incubating the culture with 1400W and applying iAβo, it does not change its effect on the frequency of mPSCs. The bar graph in (**c**) was obtained from the data recorded at time 20 ’. **d – g** NO fluorescence recordings obtained from the recorded neuron (RN) (**d** and **e**) and from adjacent neurons (NN) (**f** and **g**). Both, RN and NN cells, exhibit an increase in NO when applying iAβo in the RN neuron. This effect does not change significantly when iAβo is applied in a culture that has been pre-incubated with iNOS inhibitor. Line and bar graphs represent the average ± SEM for control (n=8), 1400W (n=7), iAβo (n=9) and iAβo + 1400W (n=10) for RN and control (n=78), 1400W (n=82), iAβo (n=75) and iAβo + 1400W (n=79) for NN. One-Way Welch’s ANOVA with Games-Howell post-hoc test for (**c**): F(3,30)=28.474, p=6.50E-9. p-values for post hoc test: Control vs. iAβo: 9.70E-7, 1400W vs. iAβo + 1400W: 3.52E-6. One-Way Welch’s ANOVA with Games-Howell post-hoc test for (**e**): F(3,30)=9.0002, p=2.10E-4. p-values for post hoc test: Control vs. iAβo: 2.41E-3, 1400W vs. iAβo

+ 1400W: 1.02E-2. One-Way Welch's ANOVA with Games-Howell post-hoc test for (**g**): $F(3,310)=21.348$, $p=1.35E-12$. p-values for post hoc test: Control vs. iAβo: 4.99E-7, 1400W vs. iAβo + 1400W: 8.97E-8. * denotes $p < 0.05$, ** $p < 0.01$, *** $p < 0.001$.

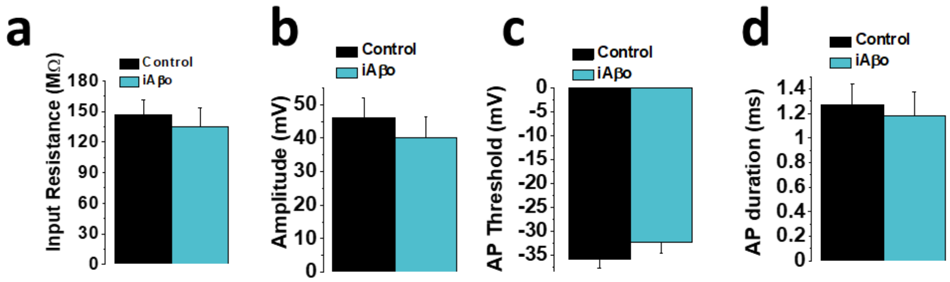

Figure 9. iAβo does not change intrinsic excitability membrane parameters in hippocampal neurons *in vivo.* a – d Quantification of input resistance and AP parameters: amplitude, duration (half-width) and threshold, all of which do not show significant differences between the conditions tested. Bar and line charts represent the average ± SEM for control (n=10) and h-iAβo (n=6) cells of at least 6 rats.

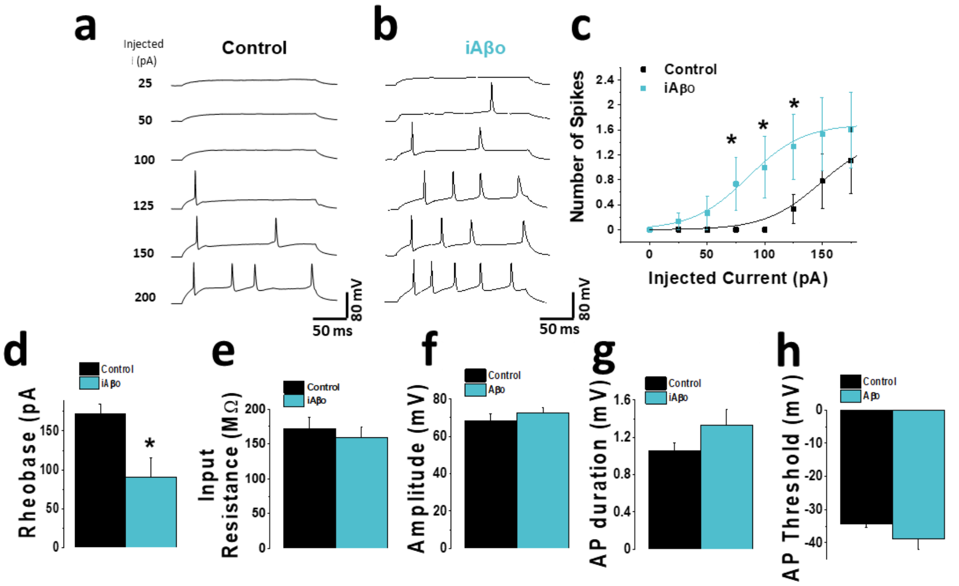

Figure 10. iAβo increased the firing of action potentials evoked by current injection in hippocampal neurons *in vitro*. a, b, Hippocampal neuron action potential (AP) recordings in the absence (**a**) and presence of 500 nM iAβo (**b**). **c,** Relationship between the number of triggered AP and the injected current intensity for the experimental conditions described previously. **d,** Rheobase constant decreased for iAβo condition. **e – h,** Quantification of input resistance and AP parameters: amplitude, duration (half-width) and threshold, all of which do not show significant differences between the conditions tested. Bar and line charts represent the average ± SEM. n=12 cells per condition. * denotes p <0.05.

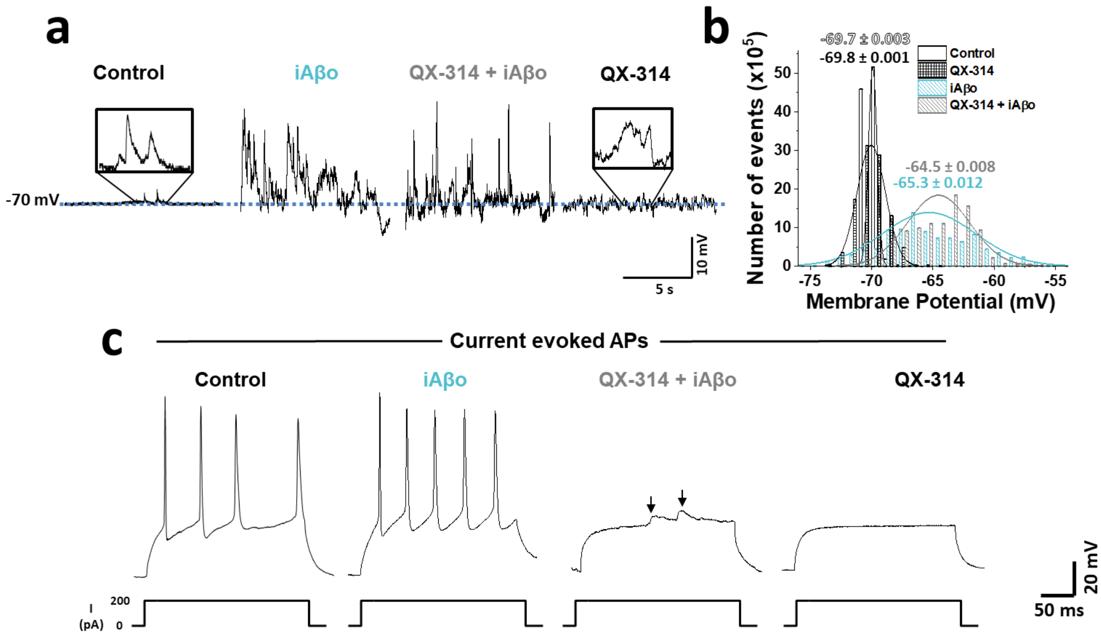

Figure 11. Intracellular blockade of voltage-regulated Na_v channels does not prevent depolarization of the membrane activated by iAβo. a, Representative recordings obtained without current injection, showing membrane potential (Vm) fluctuations under the different conditions tested. Small variations in the value of Vm are observed for control condition, which are exacerbated in the presence of iAβo 500 nM, while the co-application of iAβo with QX-314 did not diminish the intracellular effects of iAβo on Vm fluctuations. QX-314 by itself did not show any differences with respect to control conditions. **b,** Histogram showing the distribution of Vm values along with average values ± SEM in the different experimental conditions shown in **a**. **c,** Current injection experiments demonstrating that, under the control and iAβo conditions, the generation of action potentials was not inhibited, while Na_v intracellular blockade by QX-314 prevented spiking of neurons with and without iAβo. Black arrows indicate that even when Na_v was effectively blocked, depolarizing post-synaptic potentials were appreciated when iAβo was present. This did not occur for the condition with QX-314 alone. n=10 cells per condition.
